# Biodistribution of Mesenchymal Stromal Cells after Administration in Animal Models and Humans: A Systematic Review

**DOI:** 10.3390/jcm10132925

**Published:** 2021-06-29

**Authors:** Manuel Sanchez-Diaz, Maria I. Quiñones-Vico, Raquel Sanabria de la Torre, Trinidad Montero-Vílchez, Alvaro Sierra-Sánchez, Alejandro Molina-Leyva, Salvador Arias-Santiago

**Affiliations:** 1 Dermatology Department, Hospital Universitario Virgen de las Nieves, IBS Granada, 18014 Granada, Spain; manolo.94.sanchez@gmail.com (M.S.-D.); tmonterov@gmail.com (T.M.-V.); alejandromolinaleyva@gmail.com (A.M.-L.); salvadorarias@ugr.es (S.A.-S.); 2 Cellular Production Unit, Hospital Universitario Virgen de las Nieves, IBS Granada, 18014 Granada, Spain; raquelsanabriadlt@gmail.com (R.S.d.l.T.); alvarosisan@gmail.com (A.S.-S.); 3 School of Medicine, University of Granada, 18014 Granada, Spain

**Keywords:** mesenchymal stromal cell, biodistribution, cell therapy

## Abstract

Mesenchymal Stromal Cells (MSCs) are of great interest in cellular therapy. Different routes of administration of MSCs have been described both in pre-clinical and clinical reports. Knowledge about the fate of the administered cells is critical for developing MSC-based therapies. The aim of this review is to describe how MSCs are distributed after injection, using different administration routes in animal models and humans. A literature search was performed in order to consider how MSCs distribute after intravenous, intraarterial, intramuscular, intraarticular and intralesional injection into both animal models and humans. Studies addressing the biodistribution of MSCs in “in vivo” animal models and humans were included. After the search, 109 articles were included in the review. Intravenous administration of MSCs is widely used; it leads to an initial accumulation of cells in the lungs with later redistribution to the liver, spleen and kidneys. Intraarterial infusion bypasses the lungs, so MSCs distribute widely throughout the rest of the body. Intramuscular, intraarticular and intradermal administration lack systemic biodistribution. Injection into various specific organs is also described. Biodistribution of MSCs in animal models and humans appears to be similar and depends on the route of administration. More studies with standardized protocols of MSC administration could be useful in order to make results homogeneous and more comparable.

## 1. Introduction

Mesenchymal Stromal Cells (MSCs) are non-hematopoietic multipotent cells which can be isolated from different tissues from adult, perinatal and fetal samples [1,2]. Some sources are adipose tissue [3], bone marrow [4], umbilical cord Wharton’s jelly and blood [5,6], periosteum [7], skin [8], amniotic fluid [9] and the placenta [10]. These cells have the capability to differentiate into a variety of different mesenchymal lineage cells such as osteoblasts, chondrocytes, adipocytes, fibroblasts and myoblasts [2].

Since MSCs have variable phenotypes, with different expression of bio-markers depending on the source and means of isolation, as well as the tissue they come from, they cannot be considered as a homogeneous set of cells [11]. The International Society for Cellular Therapy set minimum criteria for characterizing human MSCs in order to promote a more uniform definition of MSCs. These criteria are: (a) Plastic adherence when maintained in standard culture conditions; (b) Expression of CD105, CD73 and CD90 and lack of expression of CD45, CD34, CD14, or CD11b, CD79a or CD19 and HLA-DR surface molecules; and (c) Differentiation into osteoblasts, adipocytes and chondroblasts in vitro [12].

MSCs are of great interest because of the possibility of using them as a part of therapeutic regimens in a wide variety of human diseases, e.g., rheumatic and autoimmune diseases, skin diseases and complex ulcers and wounds [13,14,15,16]. Some characteristics of MSCs are fundamental for this purpose: (a) MSCs can be obtained from adult donors and expanded in vitro without losing their immunomodulatory and differentiation potential; (b) MSCs have hypo-immunogenic properties, so allogenic sets can be used, avoiding the need for autologous cell cultures; (c) Their immunomodulatory and transdifferentiating capabilities into different cell lineages can be exploited as a novel approach to the treatment of different diseases [13,14,15,17,18,19].

Different routes of administration of MSC-based medical therapies have been described both in pre-clinical and clinical reports, and the possible differences between them, in terms of safety and efficacy, is an issue which is still under discussion [15,16,20,21,22,23]. These differences may be explained by the variable biodistribution of MSCs after their administration. The most common reported routes of administration are topical, intravenous and intraarterial, intramuscular and intralesional (including different locations e.g., skin, spinal cord, tendons).

Given the presumable importance of the different mechanisms of MSC biodistribution and their impact on the therapeutic effects, the objective of this systematic review is to describe how MSCs are distributed after their inoculation through different administration routes in animal models and humans.

## 2. Materials and Methods

### 2.1. Search Strategy

A literature search from January 2015 to April 2021 was performed using the Medline database. The following search terms were used: MSC or MESENCHYMAL STEM CELL or MESENCHYMAL STROMAL CELL or MULTIPOTENT STEM CELL or MULTIPOTENT STROMAL CELL or STEM CELL AND BIODISTRIBUTION or DISTRIBUTION.

### 2.2. Inclusion and Exclusion Criteria

The search was limited to: (a) Human or animal data; (b) In vivo studies; (c) Studies addressing the biodistribution of MSCs after any source of administration; (d) Articles written in English or Spanish. All types of epidemiological studies (clinical trials, cohort studies, case-control studies and cross-sectional studies) regarding the biodistribution of MSCs were considered.

### 2.3. Study Selection

The titles and abstracts obtained in the first search were reviewed to assess relevant studies. The full texts of all articles meeting the inclusion criteria were reviewed and their bibliographic references were checked for additional sources. Articles considered relevant were included in the analysis. Uncertainties about the inclusion or exclusion of articles were subjected to discussion until a consensus was reached.

### 2.4. Research Questions and Variables Assessed

The research questions were as follows:

- How do MSCs distribute after intravenous and intraarterial injection in animal models and humans?
- How do MSCs distribute after intramuscular injection in animal models and humans?
- How do MSCs distribute after intralesional injection in different organs and tissues in animal models and humans?
- Which cell marking techniques have recently been used in studies on humans?

The variables assessed in order to answer these questions were the model which received the MSCs (human or animal), the route of administration, the disease treated, the cell-marking technique used, the biodistribution assessment method, the time when the assessment was performed, and the outcomes regarding the biodistribution of the MSCs.

## 3. Results

**Figure 1:**
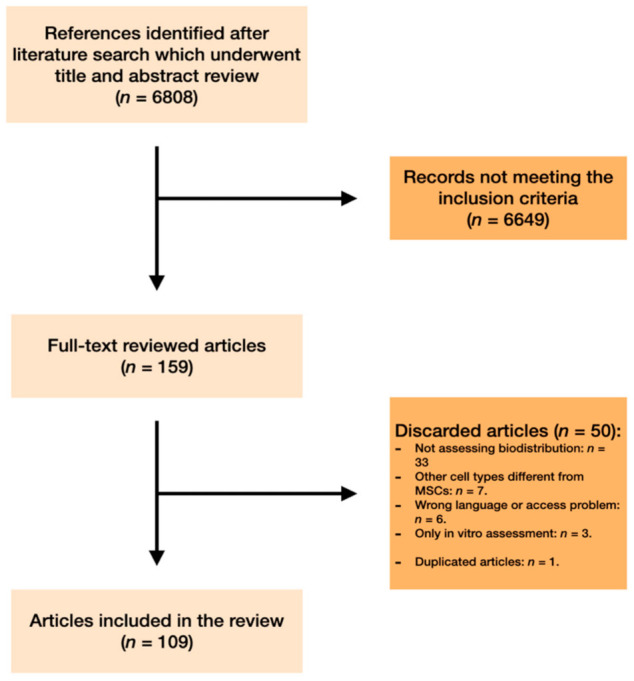
Search strategy.

An initial search found 6808 references (see Figure 1). After reviewing the titles and abstracts, 159 articles underwent full-text review. From this list, 50 articles were eventually discarded due to various issues: 33 articles did not assess biodistribution; 7 were related to other types of cells, rather than MSCs; 6 were not accessible or written in a different language; 3 only addressed the issue of in vitro MSCs; and 1 article was duplicated. Finally, 109 studies met the eligible criteria and were included in the review.

### 3.1. Biodistribution Characteristics of Mscs Depending on the Route of Administration

An overview and summary of all the information collected in this study can be seen in Table 1.

#### 3.1.1. MSC Biodistribution in Animal Models

First, the biodistribution of MSCs after their delivery or injection into animal models will be discussed. Intravenous and intraarterial infusion, intramuscular injection and a wide variety of intralesional administrations of MSCs will be addressed in this section.

#### 3.1.2. Distribution of MSCs after Intravenous Injection in Animal Models

**Figure 2:**
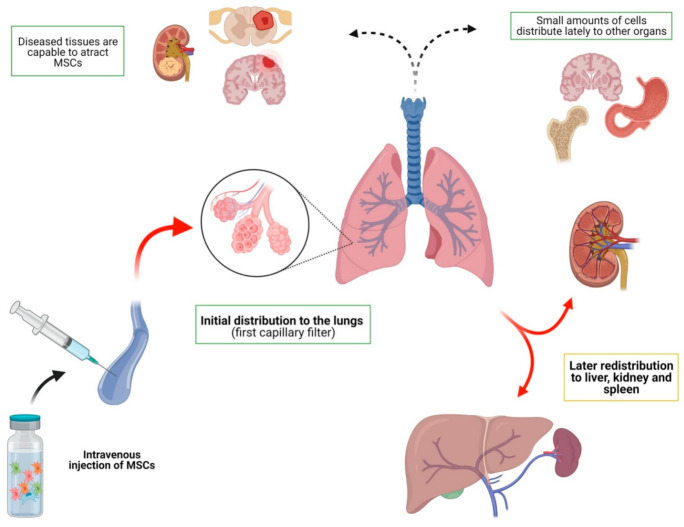
Biodistribution of MSCs after intravenous infusion. After intravenous infusion, there is initial biodistribution in the lungs. Later, most cells redistribute to the liver, kidney and spleen. Few cells can be found in other organs and tissues. In some cases, diseased tissues have been found to be capable of attracting MSCs.

Intravenous injection has emerged as the most widely used route in the various research studies. This route of administration is a simple and effective way to deliver MSCs systemically. Most of the studies discussed in this section agree on the general characteristics of how mesenchymal cells are distributed after being injected into the venous stream (Table 2, Figure 2). To begin with, some general ideas can be stated about this issue: (a) after IV injection, most cells are retained initially in the lungs, which is the first capillary filter; (b) there is later redistribution of the cells, mainly to the liver, spleen and kidney, with few MSCs redistributing to other organs; (c) in some studies, later redistribution is very limited; and (d) some pathological entities seem to alter this biodistribution pattern.

A good example of this general distribution pattern can be seen in one study assessing intravenous infusion of MSCs in a myocardial infarction model in dogs [24]. It showed high distribution during the immediate post-infusion time in the lungs, with a posterior decrease in the amount of MSCs and a later redistribution from day 1 to 7 in different tissues, mainly in the liver, spleen and kidney. A similar model of myocardial infarction in mice [25] showed early distribution in lungs but an insignificant amount of cells distributed to other organs (less than 1%). Intravenous infusion of MSCs in baboons [26], and a late evaluation of their distribution in a variety of tissues, have demonstrated a wide distribution of MSCs after a long period of time: gastrointestinal, kidney, skin, lung, thymus, and liver tissues contained MSCs. Similar results were shown in several other studies [27,28,29,30,31]. The redistribution might be explained by phagocitation of MSCs: monocytes might perform this action, and then change their immunophenotype, inducing Treg cells [32].

The alteration of the general distribution pattern in specific diseases has also been reported in several studies. Zhang et al. [33] found a significant amount of MSCs in the kidneys of rabbits with acute kidney injury. Similar results have been shown in a model of Alzheimer’s disease [34,35] with higher brain distribution of MSCs in diseased animals compared to healthy animals. This was also evidenced in another study performed on mice with cerebral tumors [36], rats and beagle dogs with spinal cord injury [37,38], and rats with intracerebral hemorrhage [39]. Moreover, acute distress respiratory syndrome or liver tumors may also affect the distribution of cells after intravenous injection [40,41]. In contrast, in a murine model of experimental autoimmune encephalomyelitis [42], MSCs were not distributed to the brain area.

Although the lungs seem to be the area MSCs mostly distribute to after intravenous injection, Schmuck et al. [43] concluded that this may be due to the lack of sensitivity of bioluminescence techniques, which are carried out in most biodistribution studies. In their study, which used a 3D cryo-imaging system, they demonstrated a higher concentration of MSCs in the liver when compared to the lungs after intravenous infusion in rats with acute lung injury. In this line, Schubert et al. [44] demonstrated a high distribution of MSCs to the lungs with bioluminescence techniques on day 1 after intravenous infusion in mice with acute kidney injury. Cells cleared on days 3 and 6. However, when RT-PCR was performed on several tissues on day 6, variable amounts of mRNA were detected in the blood, liver, kidneys and lungs. Therefore, RT-PCR could be a better option for detecting the late presence of MSCs in tissues and could be used to complement imaging techniques.

Other situations, such as the modification of MSCs or the selective infusion of MSCs into certain veins, might also affect biodistribution. Moreover, some studies have shown that modifying MSCs may lead to cells selectively targeting specific organs. The modification of specific “homing markers” or adhesion molecules can lead to targeted homing of MSCs. This has been proven by modifying MSCs to achieve specific distribution to the liver [30]. In addition, the selective intravenous delivery could lead to differences in biodistribution. For example, Li et al. [45] demonstrated that superior mesenteric vein infusion of MSCs leads to more selective and longer homing of MSCs in a model of acute liver injury when compared to intravenous and inferior vena cava delivery.

Finally, regardless of the source of administration, Fabian et al. [46] demonstrated that the age of both the recipient and the donor of MSCs seems to affect the biodistribution of the cells. The study demonstrated that old recipients and donors showed a very restricted biodistribution of MSCs in mice after 28 days (mainly in the brain cortex and spleen) whereas young receptors and donors showed a wide variety of distribution.

#### 3.1.3. Distribution of MSCs after Intraarterial Injection in Animal Models

**Figure 3:**
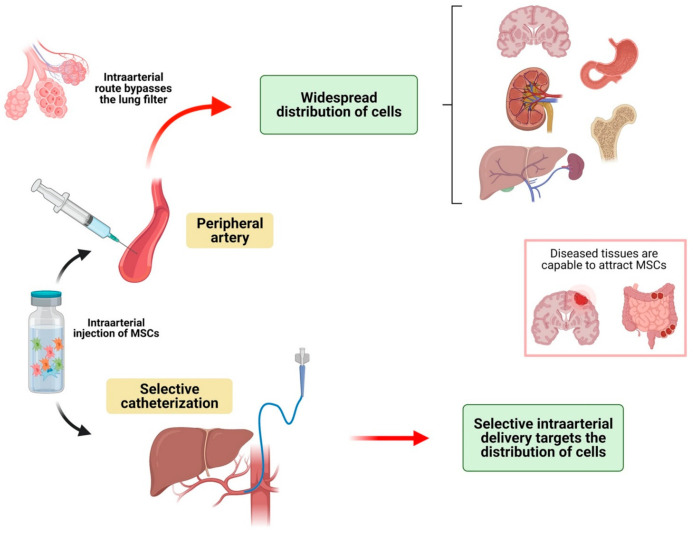
Biodistribution of MSCs after intraarterial infusion. When cells are administered into a peripheral artery, the lungs are bypassed and a wide distribution of cells is found in organs and tissues. Selective intraarterial delivery of cells targets the distribution of cells to organs which are irrigated by the cannulated artery.

Intraarterial infusion of MSCs has been used as an alternative and has also been compared to IV injection in several situations (Table 3, Figure 3). Briefly, the main characteristics of this route of administration are: (a) IA injection bypasses the pulmonary filter, so low amounts of MSCs are retained in these organs; (b) MSCs distribute more widely into the rest of the body’s organs after IA infusion compared to IV delivery; (c) like the IV route, biodistribution after IA injection might be modified by several diseases; d) selective intraarterial delivery of MSCs might be very useful for targeting diseased organs.

As an initial example of these characteristics, one study performed on pigs [22] compared intravenous and intraarterial infusion techniques. MSCs were detected using SPECT/TC imaging, which showed a lower pulmonary captation in the intraarterial group, and a relatively higher uptake in other organs such as the liver, spleen and kidney. This was also studied in an acute kidney injury model in mice [47]. In this study, a significantly higher amount of MSCs were detected in the kidneys after intraarterial infusion, especially in mice with AKI. In contrast, the vast majority of MSCs were distributed to the lungs after intravenous injection. Moreover, intracardiac injection has also been reported to be an effective delivery route. This route of administration can be considered to be equivalent to the IA route when the cells are injected into the left chambers of the heart. In fact, after intracardiac injection [48], MSCs seem to follow a similar path; widespread distribution is observed (lungs, brain, spleen, liver, kidneys).

The fact that the IA route leads to a significantly higher distribution of MSCs in peripheral organs might be an interesting characteristic when homing MSCs in the diseased area is desirable. For example, in other studies it has been proven that intraarterial injection improves distribution to the damaged cerebral areas when compared to intravenous injection [49,50,51].

Regarding the distribution of MSCs to the brain after intraarterial infusion, Cerri et al. [52] evaluated distribution to the brain of MSCs injected in the carotid artery of a Parkinson’s disease murine model. One group was treated with mannitol as a transient permeabilizing factor of the blood-brain barrier. Later assessment showed that rats not treated with mannitol had an extremely low amount of MSCs homing to the brain, whereas the group treated with mannitol showed a significantly higher amount of MSCs. Moreover, most of the cells were distributed in the ipsilateral hemisphere to the carotid used to inject them. Therefore, the use of a permeabilizing agent could be essential to allow the passage of MSCs into the brain. On the other hand, selective delivery of cells might help MSCs reach the damaged areas [51,53].

As occurred with IV injection, some pathological entities can modify the biodistribution of MSCs after IA injection. In the specific case of mice with inflammatory bowel disease, MSCs do not significantly distribute to lungs or liver but distribute mainly to the affected areas of the intestine [54]. In contrast, in a model of kidney injury, MSCs did not distribute to damaged kidneys after intracardiac injection [55]. Moreover, the dose of MSCs seems to be important when administered IA. One study showed that an increased dose of IA-administered MSCs led to a wider distribution of cells but also to a high degree of intravascular cell aggregation and mortality [56]. Thus, the dose of MSCs should be assessed before intraarterial delivery to avoid intraarterial aggregation.

The homing of MSCs to diseased tissues can be improved by selective intraarterial infusion. With this technique, MSCs are directly injected into selected arteries. This results in a greater amount of MSCs in the targeted organs. Some examples are discussed here: When MSCs are delivered directly into the renal artery, MSCs seem to distribute only in the kidneys, without systemic significant distribution, and mainly in the renal cortex [57]. Therefore, renal intraarterial MSC infusion limits off-target engraftment, leading to efficient MSC delivery to the kidneys. Similar results were found after selective intraarterial infusion into the superior mesenteric artery regarding the intestine distribution of MSCs [58], and the selective intraarterial limb infusion [59,60], with MSCs distributed in the target area and a small quantity of MSCs in the rest of the organs.

#### 3.1.4. Distribution of MSCs after Intramuscular Injection in Animal Models

**Figure 4:**
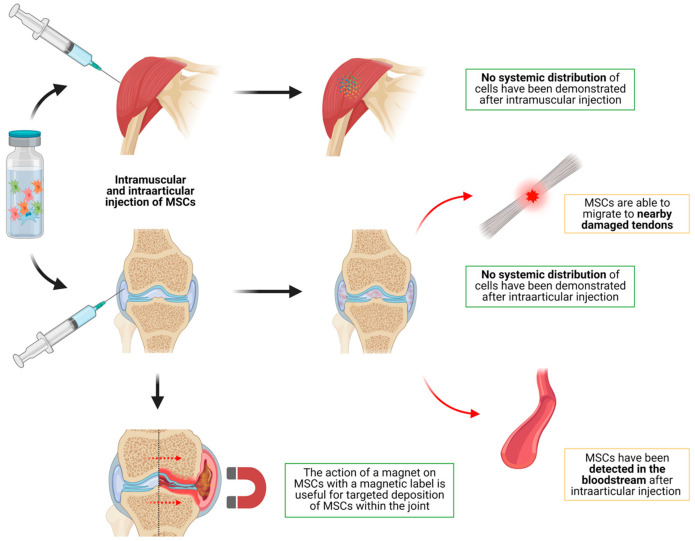
Biodistribution of MSCs after intraarticular and intramuscular injection. No systemic distribution has been demonstrated after intramuscular or intraarticular injection. After intraarticular injection, MSCs have been found to be able to migrate to nearby damaged lesions and into the bloodstream. Moreover, the use of a magnet on MSCs with a magnetic label is useful for targeted deposition of cells within the joint.

As this is widely used with classic drugs, intramuscular injection of MSCs has also been studied as a possible way to administrate MSCs (Table 4, Figure 4). As a general idea, whereas intramuscular injection of conventional drugs leads to a significant systemic distribution, MSCs injected intramuscularly do not seem to distribute to the rest of the body.

One study performed on mice to assess the sensitivity and specificity of quantitative PCR [61] for detecting MSCs showed that, 3 months after intramuscular injection of MSCs, no MSCs were detectable in any internal organ. However, DNA from MSCs was still present in the muscles where it was injected. This could suggest that MSCs do not distribute to other organs after intramuscular injection. This was in line with the findings of similar studies performed following intramuscular injection [62,63,64], with MSCs remaining at the injection site, but without MSCs distributing to organs. However, it has been demonstrated that, despite the lack of distribution of MSCs, when injected intramuscularly in a contralateral muscle to an inflamed area, MSCs are capable of reducing inflammation. This is thought to be performed by the release of soluble factors rather than the movement of the cells [65]. A recent review of intramuscular MSCs showed that, to date, no articles have found significant systemic biodistribution after intramuscular injection of MSCs [65].

#### 3.1.5. Distribution of MSCs after Intralesional Injection in Animal Models

Several different intralesional routes of administration for MSC delivery have been described. The most important routes of administration of MSCs into lesioned areas will now be addressed

- Intraarticular (IAr) delivery of MSCs (Table 4, Figure 4):

Intraarticular injection of MSCs has been widely studied in different animal models. As a general idea, IAr injection lacks systemic biodistribution, whereas it leads to a very targeted delivery of cells into the joints. This has been adequately demonstrated by studies on different mice models of healthy animals, arthritis and osteoarthritis, where it was shown that MSCs do not distribute to other organs following intraarticular injection [21,66,67,68,69]. Markides et al. [70] assessed the biodistribution of MSCs in a sheep model of osteochondral injury. After intraarticular injection, MSCs were only detected in the synovium, with a lack of MSCs within the chondral defect. Khan et al. [71] showed similar results after intratendinous injection, with no MSCs spreading from the injection site.

In contrast with that already described, some studies show an incidental distribution of MSCs. In these cases, MSCs have been shown to be present in the blood, distant zones or tendon lesions near the injection site. One study performed on a horse model of tendon lesions [72] showed that, although the vast majority of cells remained at the site where they had been injected, a small amount of MSCs could be found in blood for the first 24 h after injection, as well as in the contralateral control tendon lesions which had not been injected. Similar results were observed by Shim et al. [73]; after intraarticular injection, MSCs were detectable in blood with a peak at 8 h. No systemic distribution was observed. Moreover, other studies show that MSCs seem to be able to migrate from the joint to nearby tendinous lesions [74,75].

As occurred with the IV and IA routes, elective accumulation of MSCs in selected areas of a joint (i.e., a chondral lesion within the joint) can be achieved. MSCs must be modified by magnetic labeling. The subsequent use of a magnet during the transplantation [76] leads to the movement of the cells within the joint so they can be deposited in the target zone.

Finally, as a variant of IAr delivery, one study was performed to assess biodistribution of MSCs which were pre-loaded into bone grafts [77]. This study also showed the lack of systemic biodistribution of MSCs and the long-lasting MSCs in the graft up to 6 weeks. Similar results were found when injecting MSCs into the femoral head of pigs [78].

#### 3.1.6. Injection of MSCs into the Reproductive and Urinary System

Some studies have been found on the issue of biodistribution of MSCs after injection into the urinary and reproductive systems. In a rat model of birth-trauma injury [79], the presence of MSCs following local injection into the periurethral tissues was demonstrated up to 7 days post-injection. In this case, no tests were performed to assess the distribution to other organs after local injection. Ryu et al. [80] injected MSCs into the outer layer of the bladder in a interstitial cystitis model. It was demonstrated that cells are able to migrate from the outer layers of the bladder to the urothelium for the first 30 days after injection and to home as perivascular cells. Dou et al. [81] found that after intracavernous injection, MSCs distributed to the lower abdomen in a erectile dysfunction model in mice in the first hour. Moreover, MSCs can be found in kidney, prostate and hepatic tissues up to 7 days after injection. Finally, when injected into the ovaries, MSCs are able to distribute to the uterus, with no systemic distribution Table 5 [82].

#### 3.1.7. Injection of MSCs into the Central Nervous System

There is a wide variety of reports concerning the injection of MSCs into the central nervous system Table 6: Intrathecal, intracerebral and intraventricular injections have been described:

(a) Intrathecal injection of MSCs: After intrathecal injection, Barberini et al. [83] demonstrated that MSCs do not seem to distribute cranially (when injected in the lumbosacral area), whereas they can progress caudally (when injected in the altanto-occipital area). In this study, no MSC engraftment was demonstrated. The systemic biodistribution of the MSCs was not specifically assessed, but imaging techniques did not show the presence of MSCs in areas other than the central nervous system. In contrast, Kim et al. [84] demonstrated that MSC migration from the spine to the brain is possible in a dose-dependent manner. Quesada et al. [85] also demonstrated brain migration after intrathecal injection;
(b) Intracerebral injection of MSCs: Wang et al. [86] demonstrated that intracerebrally injected MSCs loaded with paclitaxel are capable of spreading from one cerebral hemisphere to another in a glioma model in mice in two days. These cells were found to spread from the healthy hemisphere to the glioma hemisphere and to invade the tumor. The ability of MSCs to migrate from one hemisphere to another has also been demonstrated in other studies [87]. In other reports [88,89,90,91,92,93], MSCs injected intracerebrally were detectable at the site of administration 1–3 weeks after injection, with a subsequent rapid decrease and no significant systemic distribution. Other studies [94] showed that MSCs can be detected with fluorescence and bioluminescence up to 7 weeks after transplantation;
(c) Intraventricular injection of MSCs: Some studies showed that MSCs injected into cerebral ventricles are able to migrate to large blood vessels in a brain traumatic injury model [95], and also to brain parenchyma and the spinal cord [96]. In contrast, other reports [97] demonstrate that after intraventricular infusion, MSCs do not migrate to brain parenchyma and are hardly able to migrate to the spinal cord in a model of amyotrophic lateral sclerosis.

Finally, one review showed that intranasal delivery of MSCs led to significant intracerebral migration of MSCs [98].

#### 3.1.8. Injection of MSCs into the Digestive System:

(a) Intrahepatic and intrasplenic injections have been studied in several reports as efficient delivery routes for administrating MSCs. After intrahepatic injection, Xie et al. [99] demonstrated that MSCs remain in the liver and are cleared in a short period of time, without systemic distribution. This short period of time might be in association with NK cell activation: Liu et al. [100] showed that mice with activated NK cells had a more rapid clearance of intrahepatic MSCs. Yaochite et al. [101] injected MSCs into the liver and spleen of diabetic mice. It was shown that intrasplenic MSCs were able to move to the liver whereas intrapancreatic cells remained at the site of the injection. No systemic distribution was shown and cells remained for up to 8 days. Similar results were found in another study [102], with MSCs remaining for up to 4 weeks;
(b) When injected intraperitoneally [103,104], MSCs seem to spread mostly to abdominal organs (liver, spleen and intestine) with little distribution to the lungs, heart, blood and lymph nodes. Other study shows that Wharton’s Jelly MSCs are capable of distributing to the whole body after intraperitoneal injection at days 1, 7, 14 and 21 in piglets [105].
(c) When injected in the peri-fistula area [106], MSCs do not seem to distribute systemically.

#### 3.1.9. Injection of MSCs into the Cardiovascular and Respiratory Systems

Some articles have addressed the injection of MSCs into the pericardium or the myocardium. When injected intrapericardially [107] in a myocardial infarction model, MSCs seem to distribute to ventricles and atriums, with a preference for the left ventricle. Regarding intramyocardial injection, MSCs seem to distribute initially in the myocardium, with posterior redistribution to the lungs, liver and bone [108]. Moreover, it has been demonstrated that after intramyocardial injection, if a repeated ischemia model is performed, MSCs tend to home mainly to the heart with less distribution to peripheral organs [109]. Finally, some studies were related to the injection of MSCs into the respiratory system: When injected intratracheally or intrabronchially, MSCs do not distribute systemically [110,111].

#### 3.1.10. Injection into the Skin, Subcutaneous Cellular Tissues and Lymph Nodes

Few studies have addressed the issue of biodistribution after intradermal injection of cells (Table 5). When injected into the skin of mice, Tappenbeck et al. [112] demonstrated that MSCs seem to remain in the skin and migrate to lymph nodes, without significant systemic distribution. Regarding the specific distribution in cutaneous wounds [113,114], MSCs seem to distribute with a diffuse pattern initially and later concentrate towards the wound edges. Finally, these cells seem to be engrafted with the newly developed skin tissue. No systemic distribution following intradermal injection had been reported. Only one study was performed to describe biodistribution after intranodal injection; in this study, most MSCs remain at the injection site or in the fat surrounding the injected nodes 48 h later [103], without systemic distribution of cells.

### 3.2. Biodistribution of MSCs in Humans

Only a few reports of the biodistribution of MSCs after the injection into human models have been recorded in this review (Table 7). These articles will be discussed in the following sections.

#### 3.2.1. Distribution of MSCs after Intravenous Injection in Humans

Three studies regarding the intravenous injection of MSCs into humans were identified in order to assess biodistribution. In the first study, the intravenous infusion of MSCs in patients suffering from cirrhosis showed an early (pre-48 h) distribution mainly in the lungs, with a later decrease in lung captation and a high distribution in the spleen and liver [115]. In another study on breast cancer patients, MSCs were monitored in peripheral blood after intravenous injection [116], finding a rapid clearance of MSCs in blood, with no cells detected 1 h post injection. Finally, a third article showed that when injected intravenously into a patient with hemophilia A [117], MSCs distributed early to the lungs and liver, with a progressive decrease. Distribution to the usual bleeding places was shown at 24 h.

As can be seen, biodistribution after IV injection in humans seems to be similar to that described in animal models: (a) early captation in the lungs; (b) later distribution in organs such as the spleen and liver; and (c) distribution of MSCs into target areas have been described.

#### 3.2.2. Distribution of MSCs after Intraarterial Injection in Humans

Only one study addressed the intraarterial infusion of MSCs in humans. This study was performed on 21 type 2 diabetes mellitus patients. MSCs were selectively injected intravenously or intraarterially (into the pancreaticoduodenal artery and the splenic artery). MSCs were labeled with 18-FDG and PET-TC images were used to assess biodistribution. Selective intraarterial delivery led to MSCs homing to the pancreas head (when cells were injected into the pancreaticoduodenal artery) or body (when infused into the splenic artery); whereas no MSCs were found in the pancreas in the intravenous group. This report shows that biodistribution after IA infusion of MSCs seem to be similar to biodistribution in animal models, with systemic delivery, a lack of lung trapping and the possibility of selective infusion into certain areas.

#### 3.2.3. Distribution of MSCs after Intralesional Injection in Humans

Only one study of intralesional injection of MSCs and their biodistribution was observed. Henriksson et al. [118] injected MSCs into intervertebral discs in 4 patients. Discs were explanted at 8 and 28 months post injection. Histologic examinations found the presence of MSCs in intervertebral discs after 8 months, with chondrocyte-like differentiation. No cells were found in the discs after 28 months, and no systemic distribution was assessed.

#### 3.2.4. Distribution of MSCs after Intracoronary Injection in Humans

In one study, biodistribution of MSCs after intracoronary injection was assessed. Lezaic et al. [119] injected MSCs into the coronary arteries of 35 patients with idiopathic dilated cardiomyopathy. They showed that a very low number (0–1.25%) of MSCs are retained in the myocardium, with the majority distributed to the liver, spleen and bone marrow.

#### 3.2.5. Which Cell-Marking Techniques Have Recently Been Used in Preclinical Studies?

A wide variety of cell-marking techniques have been used for preclinical studies involving cell therapy. Also, a wide variety of detection methods have been performed. Since it is not the objective of this review to address these techniques in depth, an overview of them is reviewed hereafter.

Most common cell-marking techniques can be divided into: (a) those related to the use of radionuclides; (b) those related to bioluminescence imaging systems; (c) those related to the use of magnetic resonance imaging (MRI); and (d) those related to the genetic marking of cells.

The use of radionuclides is a common technique which is useful to assess the distribution of previously marked cells in preclinical models. Some of the most common radionuclides include ^99m^Technetium-hexamethylpropyleneamine oxime (^9m^Tc-HMPAO) [21,83], or ^111^Indium-Oxine (^111^In-Oxine) [60,115]. After culture, these substances enter into the cells. Once the cells are administered, the emitted radioactivity can be detected by imaging techniques such as Single Photon Emission Computed Tomography (SPECT) or Positron Emission Tomography (PET), which allow us to track the fate of the cells. The main disadvantage of these methods are the limited duration of the radioactivity, which limits the assessment of the late distribution of cells, and the dangers related to the management of radioactive substances in the laboratory.

Bioluminescence imaging systems are based on the light which is emitted by cells which have been previously transfected with the firefly luciferase gene (*luc* gene) [99,100]. Once the cells transfected with this gene are administered to the animal, an injection of D-luciferin is performed. After D-luciferin has been administered, cells containing the specific gene fluoresce, emit light with a wavelength of 537 nm. This light can be detected by specific imaging systems to assess biodistribution. The main disadvantage of this method is that bioluminescence has limited spatial resolution and reduced tissue penetration due to the relatively weak energy of emitted photons. For these reasons, its use in large animal models is not advisable [120].

The use of superparamagnetic iron oxide nanoparticles (SPIONs) is also a useful technique to assess cell biodistribution. SPIONs are small synthetic iron particles which are coated with certain biocompatible polymers. When cells have been labeled with these particles, they are detectable by imaging techniques such as MRI techniques [121]. Given that magnetic resonance imaging is a technique that is not very accessible, the use of this method can be limited.

Finally, it is possible to label cells with specific genes that can be subsequently detected by PCR methods [26,44,105]. The main disadvantage of this cell-marking technique is that a tissue sample is required so that the distribution of cells cannot be assessed in vivo in most cases.

#### 3.2.6. Which Cell-Marking Techniques Have Recently Been Used in Studies with Humans?

The studies included in this review used different cell-marking techniques. The most common techniques are those related to the use of a radioactive labeling: MSCs can be labeled with radionuclides in vitro, and then injected into humans. Radionuclides used in the reviewed articles include ^111^Indium-Oxine (^111^In-Oxine) [115,117], 18-Fluorodeoxyglucose (^18^F-FDG) [122] and ^99m^Technetium-hexamethylpropyleneamine oxime (^9m^Tc-HMPAO) [119]. Cells are incubated in culture mediums containing these substances, which enter into the cells. Later assessment of the emitted radioactivity of these substances in the body with imaging techniques such as scintigraphy, Single Photon Emission Computed Tomography (SPECT) or Positron Emission Tomography (PET) allow us to detect the distribution of the cells in the body to be evaluated. The main disadvantage of radionuclide-based cell-marking techniques is the limited duration of the radioactivity; as the radionuclides disintegrate, the emitted signal becomes smaller and finally disappears, making it impossible to evaluate the late biodistribution of the cells administered.

Labeling cells with markers which can be detected in histologic samples is another technique used in humans. In the study reviewed [118], iron sucrose was used to label cells. This compound makes cells detectable in histologic samples. The main advantage of this kind of marker is its presumably long duration (longer than radionuclides). However, the use of histologic markers makes it necessary to perform ex vivo examination which is a limiting factor for its use in humans. The use of flow cytometry [116] to evaluate cell markers could be considered to be comparable to the use of histologic markers, and involves the extraction of biologic samples to evaluate cell distribution.

## 4. Discussion

Determining the fate of MSCs after administration is a major issue in the development of cell therapies. On one hand, as a part of their physiological functions, MSCs are able to produce several soluble substances and to modulate the immune response through different pathways; the production and induction of interleukin production and the release of microvesicles [123,124,125]. Cell interactions lead to the secretion of soluble factors and cell-to-cell contact which induces changes in the immunobiology of immune cells, such as changing the interleukin production, inducing anergy or triggering apoptosis. On the other hand, MSCs have been found to be able to differentiate into different cellular subtypes, which could play a role in regenerative medicine. Whether MSCs act by modulating the immune system or differentiating into tissular cells, understanding how and where cells are distributed after being administered by each route of administration is critical.

Intravenous injection of MSCs remains the most widely used form of administration. The widespread use of this route of administration for drugs which are used in clinical practice, and the ease of administering cells by this route, are probably the reasons why. As previously seen, IV administration might be beneficial when cell trapping in the lungs, liver or spleen is pursued, or when MSCs are capable of acting at a distance. However, intraarterial delivery might be of choice when wide systemic distribution into different tissues and organs is required. Moreover, if a targeted deposition of cells into a single organ is needed, intraarterial selective delivery could be the solution. In contrast to intravascular administration, intramuscular injection seems to lack significant systemic distribution of cells and might be preferred when the cells do not necessarily need to reach the target tissues.

Regarding intralesional administration of MSCs, there are several distribution patterns depending on the organ or tissue injected. Intraarticularly injected MSCs seem to remain in the joints, which could be of benefit when treating articular diseases. Administration into skin, lymph nodes, trachea, lungs and urinary tissues does not produce systemic distribution either, and might be useful when targeted delivery is required. In contrast, intrahepatic, intrasplenic, intracardiac and intrapericardial infusion led to a distribution of MSCs following the natural direction of the bloodstream. Moreover, the injection of cells into virtual anatomical cavities containing corporal fluids seems to produce a distribution of MSCs within the same anatomic areas: intraperitoneal, intra-cerebro-ventricular and intrathecal routes of administration make MSCs reach the organs and tissues in contact with the correspondent fluid. Finally, intracerebrally administered MSCs are able to move within the brain if induced to do so by appropriate stimuli.

## 5. Limitations and Future Studies

Although the fate of MSCs after each route of administration seems to be fairly well understood, the specific mechanisms which lead to these distribution patterns are still a matter of discussion. Moreover, as has been previously reviewed [30,46], these mechanisms might be modulated by specific factors such as the surface molecules expressed by MSCs or the age of the donors and recipients of cells. Although this is still unknown, other factors, such as the specific subtype of MSC or the donor and recipient model, could also be important. Moreover, there is great variability among different studies with respect to the exact forms of administration (e.g., the exact anatomical site injected or the concentration or volume of cells administered). The design of standardized protocols for mesenchymal cell administration could lead to less variability of results, making them more comparable.

## 6. Conclusions

Biodistribution of MSCs in animal models and humans appears to be comparable. In response to the research questions, some facts are worth noting:

(a) Intravenous administration leads to an initial accumulation of cells in the lung with later redistribution to the liver, spleen and kidneys;
(b) Intraarterial injection bypasses the pulmonary filter, so MSCs distribute more widely into the rest of the organs of the body;
(c) In both of the two previous routes of administration, selective perfusion of selected blood vessels is useful for targeting specific organs;
(d) MSCs are not distributed systemically in significant quantities after intramuscular, intraarticular, intradermal, intranodal, intratracheal, intrapulmonary and intraurinary tissue administration;
(e) The injection into specific organs, such as the liver, spleen, pericardium or heart leads to a distribution of MSCs following the direction of the natural bloodstream;
(f) The injection into anatomical cavities containing body fluids (cerebral ventricles, subarachnoid space and peritoneum) leads to a distribution of MSCs in tissues which are in contact with the fluid;
(g) MSCs injected intracerebrally seem to be able to migrate within the central nervous system.

**Table 2:** Biodistribution after IV administration of MSCs in animal models.

Article	Model	Disease (Number of Animals)	Route of Administration (Source of Cells)	Cell-Marking Technique	Detection Time and Outcome	Comments
Krueger et al. [126] (2018)	Adult baboons [26]	Lethal total body irradiation (3 animals)	Intravenous (autogenic and allogenic MSCs)	Genetic transduction with green fluorescent protein retroviral construct, which was later evaluated by PCR.	Necropsies were performed between 9 and 21 months following MSC infusion. Several tissues were found to have MSCs: Gastrointestinal, kidney, skin, lung, thymus, and liver.	Gastrointestinal tissues had the highest MSCs concentration. MSCs distribute to a wide variety of tissues following systemic administration.
	Mongrel dogs [24]	Miocardial infarction (7 animals)	Intravenous (allogenic MSCs)	^111^In oxine–labeled MSCs colabeled with ferumoxides–poly-l-lysine. Single-photon emission CT (SPECT) and x-ray CT (SPECT/CT) and MRI studies were used to evaluate the distribution.	Imaging was performed immediately after injection and at multiple time points between 1 and 7 days after infusion. Early imaging showed a high distribution to lungs, which later decreased drastically. After day 1, MSCs distributed from lungs to different organs (kidney, bone marrow, liver, spleen) and also to the infarcted area.	A high and early distribution to lungs is showed, with a progressive decrease of MSCs and a later redistribution to a wide variety of tissues.
	Mice [25]	Miocardial infarction (number unknown)	Intravenous (xenogenic MSCs—human MSCs)	Human MSCs were infused, Quantitative assays for human DNA and mRNA were used to evaluate the distribution,	Tests were done at 15 min, and up to 100 h post infusion. Early distribution to the lungs was detected (15 min). Later distribution to other organs was insignificant: less than 1% of cells was detected in any other organ after 48 h.	Authors conclude that effects of intravenous MSCs might be due to soluble mediators rather than engraftment of MSCs in target tissues.
Mello et al. [39] (2020)	Rats	Intracerebral hemorrhage	Intravenous (xenogenic MSCs—human MSCs)	^99m^Tc was used to label MSCs. Scintigraphy and radioactivity measurements (cerebral hemispheres, heart, lungs, liver, kidneys, intestines, and spleen) were performed to assess biodistribution.	Scintigraphy was performed 2 h after cell injection and ex vivo radioactivity was evaluated 24 h after cell transplantation. MSCs were mainly distributed to the lungs, kidneys, spleen and liver. Brain captation was low but it was relatively higher in the damaged hemisphere.	
Patrick et al. [127] (2020)	Mice	Lung cancer	Intravenous (xenogenic MSCs—human MSCs)	^89^Zr-oxine and luciferase were used to label MSCs. PET-CT, bioluminescence and ex vivo radioactivity measures were used to assess biodistribution.	PET-CT at 1 h and 1, 2, and 7 days post-injection. At 7 days, radioactivity was measured from ex vivo organs. The majority of signal (60%) was found in the lung at 1 h before decreasing, while liver signal increased. From 1 to 7 days post-injection, the proportion of the ^89^Zr signal in the lung fell further from 24.6%.	
Wuttisarnwattana et al. [128] (2020)	Mice	Bone marrow transplanted animals	Intravenous (xenogenic MSCs—human MSCs)	Red quantum dots were used to label MSCs. Ex vivo cryo-imaging was performed to assess biodistribution in different tissues (lung, liver, spleen, kidneys, bone marrow).	Animal sacrifice was performed at different time points following stem cell infusion (24, 48, 72 h). Initially, MSCs were found as clusters in the lung and eventually dissociated to single cells and redistributed to other organs within 72 h, mainly to the spleen and liver.	
De White et al. [32] (2018)	Mice	Healthy animals (number unknown)	Intravenous (xenogenic MSCs—human MSCs)	Qtracker 605 beads and Hoechst33342, which labelled alive and dead cells, respectively. Anatomical and molecular fluorescence videos were generated with CryoViz Technology. Blood tests were performed to analyze phagocytosis.	Necropsies were performed at 5 min, 24 h and 72 h post-infusion. Early accumulation of MSCs in the lungs (5 min) was demonstrated. MSCs were phagocytized in the lungs and redistributed to liver within the monocytes at 24 and 72 h. Monocytes change their immunophenotype after phagocyting MSCs, and induce Treg cells.	Authors conclude that the action of MSCs in many organs may be due to the phagocytosis of MSCs by monocytes and the later change in their phenotype, which leads to the induction of Treg cells.
Ehrhart et al. [35] (2016)	Mice and rats	Alzheimer’s disease model	Intravenous (xenogenic MSCs—human MSCs)	Human MSCs were used. Tisular PCR analyses (blood, bone marrow, brain, spinal cord, spleen, kidney, liver, heart, lung, gonad) were used to assess biodistribution.	Harvesting of tissues was performed at 24 h, 7 days, and 30 days after injection. MSCs were broadly detected both in the brain and several peripheral organs, including the liver, kidney, and bone marrow, of both species, starting within 7 days and continuing up to 30 days post-transplantation.	
Tang et al. [129] (2016)	Rats	Cirrhosis rats (splenectomized)	Intravenous (allogenic MSCs)	Qtracker705 nanoparticle-labelled MSCs were infused. Fluorescence imaging was performed to assess biodistribution.	Images were taken at 2 h and 5 days after cell infusion. Splenectomy improved the homing of MSCs in the liver when compared to non-splenectomy group.	
Cao et al. [130] (2016)	Rats	Healthy animals	Intravenous (allogenic MSCs)	Luciferase and green fluorescent protein were used to label MSCs. Bioluminescence imaging, ex vivo organ imaging, immunohisto-chemistry (IHC), and RT-PCR were used to assess biodistribution.	Images were taken up to 1 month. After that, histological analysis was performed. MSCs were detected initially in the lungs with subsequent distribution to liver, kidneys and other abdominal organs. The dorsal skin was also detected to have MSCs. The signals disappeared at day 14.	
Zhou et al. [131] (2015)	Rats	Hepatic fibrosis	Selective intravenous (superior mesenteric vein) (allogenic MSCs)	MSCs were double-labeled with superparamagnetic iron oxide and green fluorescent protein. MRI, histology and qPCR tests were used to assess biodistribution.	MR imaging of the liver was carried out before and 1, 3, 7 and 12 days after injection. Liver, lung, kidney, muscle and heart tissues were harvested at 1, 7, 15 and 42 days after cell injection. Dual-labeled MSCs were retained in the fibrotic liver of rats. SPIO particles and EGFP-labeled BMSCs showed a different tissue distribution pattern in rats with liver fibrosis at 42 days after transplantation.	SPIO-based MR imaging may not be suitable for long-term tracking of transplanted BMSCs in vivo.
Kim et al. [36] (2015)	Mice (athymic)	Brain tumor	Intravenous and intracerebral (xenogenic MSCs—human MSCs)	MSCs were labeled with near-infrared fluorescent dye. Bioluminescence and fluorescence imaging, qPCR and histologic examinations were performed.	Imaging techniques were performed at 1 and 4 h, 1, 7, 14 and 21 days. MSCs resided predominantly in the lung up to day 1 and the signal intensity decreased over time. Many cells moved from the lung toward other organs (liver and spleen) after day 1, and the signal remained stable in these regions for 14 days. From day 1 to day 14, MSCs were clearly detectable in the tumor area.	
Kim et al. [38] (2015)	Beagle dogs	Spinal cord injury	Intravenous (allogenic MSCs)	MSCs were labeled with green fluorescent protein. Ex vivo bioluminescence was used to assess biodistribution.	Ex vivo examination was performed 7 days after injection. The green fluorescent protein-expressing AD-MSCs were clearly detected in the lung, spleen, and injured spinal cord; however, these cells were not detected in the liver and un-injured spinal cord.	
Li et al. [45] (2015)	Mice	Acute liver injury	Selective intravenous: Inferior vena cava (IVC), superior mesenteric vein (SMV) and intrahepatic (IH) injection. (allogenic MSCs)	MSCs were labeled with luciferase. Bioluminiscece images were used to assess biodistribution.	Images were taken at 3 h, and at 1, 3, 7, 10, 14 and 21 days. After IVC infusion, MSCs were quickly trapped inside the lungs, and no detectable homing to the liver was observed. By IH injection, lung entrapment was bypassed, but MSCs-R distribution was only localized in the injection region of the liver. After SMV infusion, MSCs-R were dispersedly distributed and stayed as long as 7-day post-transplantation in the liver.	SMV is the optimal MSCs delivery route for liver disease.
Zhang et al. [33] (2015)	Rabbit	Acute ischemic kidney injury	Intravenous (allogenic MSCs)	MSCs were labeled with SPION particles. MRI images and histological analysis were used to assess biodistribution	Images and histological analysis were taken at 1, 3, 5 and 8 days. MSCs were detected up to 8 days, with a maximum amount of cells at day 3. No systemic distribution was assessed.	
Schmuck et al. [43] (2016)	Sprague-Dawley rats	Acute lung injury (12 animals)	Intravenous (xenogenic MSCs—human MSCs)	MSCs were labeled with QTracker65. 3D cryo-imaging of lungs, liver, spleen, heart, kidney, testis, and intestine was performed to assess biodistribution.	Tissue samples were collected and analyzed at 60, 120 and 240 min and 2, 4 and 8 days after infusion. Distribution up to 240 min was detected mostly in liver, and also in lungs and spleen. The number of cells detected at 2, 4, and 8 days was less than 0.06% of the total cells infused on day 0 and were mainly distributed also in lungs, liver and spleen but relatively higher captation was seen in the rest of the tissues studied.	Authors conclude that studies using bioluminescence to track cells underestimate cell retention in the liver because of its high tissue absorption coefficient
Li et al. [27] (2018)	Rats	Silicosis (54 animals)	Intravenous (allogenic MSCs)	MSCs were labelled with 1,1′-dioctadecyltetramethyl indotricarbocyanine iodide. Fluorescence imaging was performed to assess biodistribution.	Images were taken 1 h, 6 h, 24 h, 3 days, 15 days, and 30 days after injection both in vivo and ex vivo. MSCs distributed mostly in liver and lungs, with a peak at 6 h, and a dramatic decrease by day 3. At day 30, no MSCs were detected.	Distribution in lungs was significantly higher in rats with damaged lungs compared to healthy rats.
Park et al. [34] (2018)	Mice	Alzeimer’s disease (53 animals)	Intravenous (allogenic MSCs)	MSCs were ^111^In-tropolone labeled. Imaging with SPECT (in vivo) and gamma-counter (ex vivo) was performed to assess biodistribution.	Imaging and gamma-counter studies were performed at 24 h and 48 h post infusion. In Alzheimer’s model, brain uptake of MSCs was significantly higher than in healthy animals. In both groups, MSCs distributed mainly to lungs, liver and spleen.	Distribution to brain seem to be higher in Alzheimer’s models.
Leibacher et al. [28] (2017)	Mice	Healthy animals (number unknown)	Intravenous (xenogenic MSCs—Human MSCs)	Human MSCs were injected and PCR techniques were used to assess biodistribution by searching for SRY sequences.	Ex vivo assessment was performed at 5 min, 30 min, 2 h, 6 h, and 24 h. The majority of injected MSCs were detected by qPCR in the lungs 5 min after transplantation, whereas <0.1% were detected in other tissues over 24 h	After intravenous injection, most cells distribute to lungs.
Yun et al. [31] (2016)	Rats	Acute liver injury	Intravenous (xenogenic MSCs—Human MSCs)	Human MSCs were injected and PCR techniques were used to assess biodistribution.	Mice were euthanized at 1, 3, 12, or 24 h and at 1, 4, or 13 weeks post injection. MSCs were detected soon in the lungs and disappeared before 1 week post injection. Then, MSCs were found mainly in the liver. No MSCs were found in other tissues (testis, ovary, spleen, pancreas, kidney, adrenal gland, thymus, and brain).	
Abramowski et al. [42] (2016)	Mice	Experimental autoimmune encephalomyelitis model (number unknown)	Intravenous (allogenic MSCs)	MSCs were injected and a variety of techniques, including magnetic resonance imaging, immunohistochemistry, fluorescence in-situ hybridization, and quantitative polymerase chain were performed to assess biodistribution.	Assessment was focalized in the brain area. No evidence for immediate migration of infused MSC into the central nervous system of treated mice was found.	
Kim et al. [30] (2016)	Rats	Healthy rats	Intravenous (allogenic MSCs)	MSCs were surface-modified with HA—wheat germ agglutinin (WGA) conjugate for targeted systemic delivery of MSCs to the liver and labeled with fluorescent dyes. Histologic examinations were performed.	Assessment was performed at 4 h post injection. Lungs and livers were collected. HA-WGA-MSCs had a greater distribution to the liver when compared to control MSCs, which were mainly trapped in the lungs.	HA-WGA conjugate has great potential to deliver MSCs to the liver efficiently within a short time and to reduce the entrapment of MSCs in the lung.
Lu et al. [40] (2016)	Mice	Acute distress respiratory syndrome model	Intravenous (allogenic MSCs)	Fluorescein isothiocyanate– dextran was used to label MSCs. Histological analyses and qPCR were used to assess biodistribution.	Assessment was performed immediately after cell injection, 2, 24, and 48 h later. Lung, heart, spleen, kidney, brain, and liver were collected. MSCs accumulated mainly in the lungs of control and diseased mice, with minor amounts distributed to other organs up to 2 h. Diseased animals showed less early distribution to lungs and higher distribution to the rest of the organs when compared to healthy animals.	Acute distress respiratory syndrome might lessen the pulmonary capillary occlusion by MSCs immediately following cell delivery while facilitating pulmonary retention of the cells.
Fabian et al. [46] (2017)	Young and old mice	Alzheimer disease (unknown number)	Intravenous (syngenic MSCs)	Histologic and genetic tests (PCR) were performed to evaluate MSCs distribution.	Genetic tests and histology were assessed after 28 days. Transplantation of MSCs obtained from old mice showed biodistribution only in the blood and spleen in both young and old mice. MSCs obtained from young mice showed a wide distribution in young receptors (lung, axillary lymph nodes, blood, kidney, bone marrow, spleen, liver, heart, and brain cortex). In contrast, these cells showed distribution only in the brain cortex in old mice.	Authors conclude that aging of both the recipient and the donor MSCs used attenuates transplantation efficiency.
Ohta et al. [37] (2017)	Rats	Spinal cord injury	Intravenous (allogenic MSCs)	MSCs were labeled with ^3^H-thymidine. Histologic and radioactivity examination of the spinal cord segment containing the damaged region, blood and target organs were harvested.	After 3, 24 and 48 h, organs were collected and radioactivity measured. The highest radioactivity was detected in the lungs 3 h after infusion, while radioactivity in the injured spinal cord was much lower. However, brain radioactivity was lower than damaged spinal cord.	MSCs distribute to the injured spinal crod.
Liu et al. [29] (2018)	Mice	Acute lung injury	Intravenous (xenogenic MSCs—Human MSCs)	MSCs were labeled with fluorophore Cy7. Histology was performed to assess biodistribution.	Ex vivo assessment of lungs, heart, spleen, kidneys and liver was performed at 30 min, 1 day, 3 days and 7 days following injection. MSCs distributed to the lungs up to day 1; and to the liver up to day 3, with progressive subsequent decrease. No significant distribution was observed to heart, spleen and kidneys	
Qin et al. [41] (2018)	Rabbits	Liver tumors	Intravenous (allogenic MSCs)	MSCs were colabeled with superparamagnetic iron oxide (SPIO) particles and 4′,6-diamidino-2-phenylindole (DAPI). MRI and histologic examination were performed.	MRI was performed at days 0, 3, 7 and 14 after cells transplantation. Histological analyses were performed immediately after the MRI examination. MSCs were detected in the liver tumors, rather than the non-tumor liver tissue and other organs. At day 3, MSCs were mainly in the central part of the tumor, showing a posterior distribution in the periphery.	MSCs distribute mainly to the damaged liver when injected intravenously.
Leibacher and Henschler [132] (2016)	Wistar rats [133]	Transient cerebral ischemia (25 animals)	Intravenous and intraarterial (allogenic MSCs)	Feridex (Berlex Imaging) mixed with the transfection agent poly-l-lysine. Later evaluation with MRI and necropsies.	Imaging was performed before and after the infusion (2 to 24 h after). After intraarterial infusion, MSCs were detected in the brain of the rats. After intravenous infusion, no MSCs were detected in the brain.	Authors conclude that MSCs may engraft in peripheral tissues after intraarterial infusion. Intravenous infusion might not be quite effective to deliver MSCs to peripheral tissues.
	Mice [47]	Healthy animals and acute kidney injury (AKI) model (Unknown number)	Intravenous and intraarterial. (allogenic MSCs)	Transfection with luciferase-neomycin phosphotransferase construct. Later evaluation with Xenogen IVIS 100 imaging system.	Imaging was performed immediately after infusion, at 24 h, 72 h and 7 days. Intravenous infusion led to a majority of cells distributing to lungs. Intraarterial infusion lacked pulmonary retention and caused distribution to kidneys, especially in AKI mice. MSCs gradually disappeared after 24 h.	Intraarterial infusion might be adequate when treating kidney conditions.
Schubert et al. [44] (2018)	Mice	Acute kidney injury model (Unknown number)	Intravenous. (autogenic MSCs)	MSCs from luciferase transgenic mice. Evaluation was performed with bioluminescence imaging and RT-PCR.	Imaging was performed on days 1, 3 and 6. RT-PCR was performed in kidney, lung, liver tissue and blood on day 6. Bioluminescence showed a high distribution of MSCs to lungs on day 1, which disappeared on days 3 and 6. RT-PCR on day 6 showed variables amounts of MSCs-mRNA in blood, liver and kidneys	RT-PCR seems to be a more sensitive technique to demonstrate the late presence of MSCs in different tissues when compared to bioluminescence.
Nakada and Kuroki [62]	Mice	Healthy animals (Unknown number)	Intravenous and intramuscular (allogenic MSCs)	MSCs were labelled with chromium. Laser ablation inductively coupled plasma imaging mass spectrometry (LAICP-IMS) was used to assess biodistribution,	Detection time is not recorded. After intramuscular injection, MSCs remain in the muscular tissue. After intravenous injection, MSCs are detected in the lungs.	Authors conclude that chromium labelling could be a promising technique.
Mäkelä et al. [22] (2015)	Pigs	Healthy animals (12 animals)	Intravenous and intraarterial (autogenic and allogenic MSCs)	^99m^Tc- hydroxymethyl-propylene-amine-oxime. Evaluation was performed with SPECT/TC. Biopsies were also performed.	Imaging was performed 8 h later. Intravenous infusion led to a high distribution of MSCs into the lungs. Intraarterial infusion decreased the deposition in the lungs and increased the uptake in other organs, specially the liver and kidneys.	Intraarterial infusion might improve the distribution to peripheral tissues and may avoid pulmonary retention.
Wang et al. [134] (2015)	Mice	Bone marrow transplanted animals	Intravenous and intraarterial (xenogenic MSCs—Human MSCs)	^99m^Tc- hydroxymethyl-propylene-amine-oxime and luciferase. Bioluminescence, scintigraphy and histologic examination were used to assess biodistribution.	Bioluminescence was performed at 30 min, 24 h, 48 h, 96 h and once a week for up to two month. Scintigraphic imaging and X-ray imaging were performed at 5 h, 10 h and 1 d after injection. After 2 months, animals were sacrificed and ex vivo histology was performed. After intraarterial injection persistent whole–body MSC distribution in allo-trasplant recipients was shown, while MSCs were rapidly cleared in the syngeneic animals within one week. In contrast, intravenous injected MSCs were mainly seen in the lungs with fewer cells traveling to other organs.	
Silachev et al. [49] (2016)	Rats	Traumatic brain injury model	Intravenous and intraarterial (allogenic MSCs)	^9m^Tc and iron microparticles labelled MSCs. Evaluation was performed with SPECT/TC, MRI and histology.	Evaluation was performed at 1 h and 16 h after trasplantateion. After intravenous injection, MSCs distributed to lung, kidney, and partially in the liver and bladder, with progressive decrease to 16 h. After intraarterial injection, MSCs distributed significantly to damaged hemisphere.	Intraarterial injection improves the distribution to the damaged cerebral area.
Cao et al. [50] (2018)	Rats	Orthotopic glioma model	Intravenous, intraarterial and intratumoral (allogenic MSCs)	MSCs were transduced to express ferritin heavy chain and green fluorescent protein. MRI and histology evaluations were performed.	MRI was performed at days 0, 1, 3, 5, 7 and 9 after cell injection. Histological analysis was performed at days 8, 12 and 18. Intravenous injection did not lead to accumulation of MSCs in the tumor. However, intralesional and intraarterial injections showed a rapid accumulation of MSCs in the core of the tumor with a gradual decrease of the cells in the zone.	Intravenous injections does not lead to MSCs migration to central nervous system tumors, whereas intraarterial and intralesional injections do.
Taylor et al. [55] (2020)	Mice	Renal injury model	Intravenous and intracardiac (allogenic MSCs)	MSCs were labelled with luciferase and SPIO. MRI and bioluminescence were used to assess biodistribution.	Images were taken up to 2 days after injection. Following intravenous administration, no MSCs were detected in the kidneys, irrespective of whether the mice had been subjected to renal injury. After intracardiac injection, MSCs transiently populated the kidneys, but no preferential homing or persistence was observed in injured renal tissue.	
Scarfe et al. [48] (2018)	Mice	Healthy animals (unknown number)	Intravenous and intracardiac (left ventricle) (allogenic MSCs and xenogenic MSCs—human MSCs)	MSCs were labelled with luciferase (Luc) or a bicistronic construct of Luc and ZsGreen for bioluminescence imaging. For MR tracking, cells were labelled with diethylaminoethyl-dextran-coated SPIONs.	In vivo biodistribution of cells was monitored by BLI immediately after cell administration and at multiple time points up to 30 day. Ex vivo MRI at baseline and up to 2 days post administration. Intravenous MSCs distributed mainly to the lungs. Intracardiac MSCs distributed to the brain, heart, lungs, kidney, spleen and liver, with also a majority of cells distributing to the lungs.	Intracardiac injection led to a wide distribution of MSCs to peripheral organs.

**Table 3:** Biodistribution after IA administration of MSCs in animal models.

Article	Model	Disease (Number of Animals)	Route of Administration (Source of Cells)	Cell-Marking Technique	Detection Time and Outcome	Comments
Khabbal et al. [51] (2015)	Rats	Ischemic stroke model	Intraarterial (external carotid) (allogenic MSCs and xenogenic MSCs—Human MSCs)	MSCs were labeled with ^99m^Tc. Whole body SPECT images and ex vivo radioactivity measures were used to assess biodistribution.	SPECT images were acquired 20 min, 3 h, and 6 h postinjection, after which rats were sacrificed for ex vivo examinations. The majority of the cells were located in the brain and especially in the ipsilateral hemisphere immediately after cell infusion. This was followed by fast disappearance. At the same time, the radioactivity signal increased in the spleen, kidney, and liver.	Human MSCs had faster clearance from the brain than rats MSCs.
Fukuda et al. [56] (2015)	Rats	Ischemic stroke model	Intraarterial (Common carotid artery) (xenogenic MSCs—human MSCs)	Human MSCs were used and labeled with PKH26. Bioluminescence and anti-human vimentin antibodies were used to assess biodistribution of MSCs in ex vivo histological analysis.	Examinations were performed 24 h post infusion. MSCs were widely distributed throughout the cortex and striatum of the ipsilateral hemisphere at 24 h after transplantation of MSCs.	
Cerri et al. [52] (2015)	Wistar rats	Parkinson’s disease (unknown number)	Intraarterial. (One group also received mannitol to transiently permeabilize the blood-brain barrier). (allogenic MSCs)	MSCs were double-labelled: CellVue NIR815 Kit for Membrane Labeling (Polyscience, Warrington, PA, http://www.polysciences.com) (accessed on 25 June 2021) and lipophilic red fluorescence dye PKH26. Later histological examinations assessed the distribution of MSCs within the brain.	Necropsies were performed 7 and 28 days after infusion of MSCs. Rats not treated with mannitol showed a very low number of MSCs in the brain at 7 and 28 days post-infusion. Rats treated with mannitol showed a significantly higher number of MSCs within the brain. At day 7, most of MSCs were in the blood vessels, whereas at day 28, most of MSCs were in the parenchyma. Most of MSCs distributed in the same lateral hemisphere where the infusion took place. A strong MSCs signal in the lungs and spleen up to 28 days after infusion was detected.	Authors conclude that the use of a permeabilizing agent is essential to allow passage of MSCs across the blood-brain barrier. A significant number of infused cells accumulated in the peripheral organs (liver, lungs).
Jin et al. [135] (2016)	Beagle dogs	Osteonecrosis of the femoral head	Intraarterial (autogenic MSCs)	MSCs were labeled with 5-bromo-2-deoxyuridin. Histologic examinations (right femoral head, heart, lung, liver, spleen, kidney, gallbladder, small bowel, pancreas, prostate, and testicle) were performed to assess biodistribution.	Histologic examinations were performed 8 weeks after cell infusion. Organs had uneven distribution of MSCs: Heart, liver, gallbladder, kidney and stomach had the major quantity of MSCs.	
Arnberg et al. [58] (2016)	Rabbit	Healthy rabbits	Intraarterial infusion (superior mesenteric artery) and intravenous (xenogenic MSCs—Human MSCs)	MSCs were labeled with ^11^In-oxinate. SPECT-TC images were used to assess biodistribution.	SPECT-TC was performed at 6 h and at 1, 2, and 5 days post infusion. Intravenous administration resulted in early and long distribution of MSCs to the lungs. In contrast, selective intraarterial injections resulted in MSCs distribution in the intestine and in the liver.	Selective intraarterial delivery could improve the results in treating some localized diseases.
Espinosa et al. [59] (2016)	Horses	Healthy horses	Intraarterial selective infusion (median artery) (allogenic MSCs)	MSCs were labeled with ^99m^Tc-HMPAO. Scintigraphic images were taken to assess biodistribution.	Images were taken at the time of injection and at 1, 6, and 24 h postinjection. Homogeneous distribution of radiolabeled MSC was observed through the entire distal limb, including within the hoof. Systemic biodistribution was not assessed.	
Sierra-Parraga et al. [57] (2019)	Pigs	Renal ischemia-reperfusion injury. (unknown number).	Intraarterial infusion (renal artery) (allogenic MSCs)	MSCs were labelled with fluorescent compunds. Flow cytometry and genetic tests (PCR) were done in blood and tissues.	Samples were collected 30 min and 8 h after infusion. After infusion, a minor number of MSCs left the kidney through the renal vein, and no MSCs were identified in arterial blood. A low percentage of the infused MSCs were present in the kidney 14 days after administration. Most of MSCs were trapped in the renal cortex.	Renal intra-arterial MSC infusion seem to limit off-target engraftment, leading to efficient MSC delivery to the kidney.
Barthélémy et al. [60] (2020)	Golden Retriever Dogs	Duchenne muscular dystrophy model	Intraarterial (femoral artery) (not stated)	MSCs were labeled with ^111^In-oxine. Scintigraphy was performed to assess biodistribution.	Scintigraphic images were taken immediately after injection and at 1, 2, 24, 48 h and 1 week. Immediately after injection, MSCs were trapped in the capillary network of the limb and in the lungs. Subsequently, MSCs were also mainly in the injected limb, with a decrease in the lung captation and a relative increase in the liver captation.	

**Table 4:** Biodistribution after intramuscular and intraarticular administration of MSCs in animal models.

Article	Model	Disease (Number of Animals)	Route of Administration (Source of Cells)	Cell-Marking Technique	Detection Time and Outcome	Comments
Hamidian Jahromi et al. [63] (2017)	Mice	Carrageenan-induced plantar inflammation	Intramuscular (contralateral to plantar inflammation) (xenogenic MSCs—Human MSCs)	MSCs were labelled with Gaussia Luciferase. Bioluminescence imaging, qPCR and histology techniques were used to assess biodistribution.	Bioluminescence was performed at 24 h, 48 h and up to 33 days. No MSCs were found to distribute to other organs. MSCs were detectable in the muscle up to 33 days after injection.	MSCs were able to reduce the contralateral inflammation and to lower the TNF-alfa serum levels without distributing systemically.
Creane et al. [61] (2017)	Mice	Healthy mice (10 animals)	Intramuscular (xenogenic MSCs—Human MSCs)	Human MSCs were injected and quantitative PCR for Alu sequences was performed in different tissue samples.	Ex vivo analysis was performed 3 months after injection. No MSCs were detected in any organ, including heart, lung, brain, liver, kidney and spleen. MSCs were detected in the thigh and calf samples, where MSCs were injected.	Intramuscular MSCs do not seem to remain viable and/or distribute 3 months after injection.
Hamidian Jahromi et al. [65] (2019)	Rats and mice (Review)	Different diseases	Intramuscular (different sources of MSCs)	Different techniques.	MSCs do not seem to distribute after intramuscular injection. MSCs seem to remain or spread locally, without systemic biodistribution.	Intramuscular MSCs do not seem to distribute systemically.
Cai et al. [64] (2017)	Rats	Healthy rats	Intramuscular (allogenic MSCs)	Melanin-based gadolinium3+ (Gd3+)-chelate nanoparticles were used to label MSCs. MRI was used to assess biodistribution.	MRI was performed on days 1, 4, 7, 14, 21, and 28. MSCs were found in the muscle up to 28 days after injection. No systemic biodistribution was observed.	Intramuscular MSCs do not seem to distribute systemically
Markides et al. [70] (2019)	Sheep	Osteochondral injury	Intraarticular (autogenic MSCs)	MSCs were labelled with Nanomag, and using a cell-penetrating technique, glycosaminoglycan-binding enhanced transduction (GET). Evaluation was performed with ex vivo MRI and histologic tests.	Ex vivo MRI and histology was performed 7 days after injection. MSCs were detected in the synovium, and not in the osteochondral defect.	MSCs are capable to home in the synovium, whereas they do not seem to be able to enter the joint to reach the osteochondral defect.
Yang et al. [74] (2019)	Mice	Supraspinatus tendon tear	Intraarticular (allogenic MSCs)	MSCs were labeled with quantum dots with near-infrared properties. Near-infrared fluorescence imaging was used to assess biodistribution.	Imaging was performed at days 1, 3, 7, 11, 14, and 17. MSCs did not distribute systemically. MSCs tended to migrate from the joint to the place of the lesion.	
Satué et al. [75] (2019)	Rats	Patellofemoral cartilage defect	Intraarticular (allogenic MSCs)	MSCs expressing heat stable human placental alkaline phosphatase were used. Histological and immuno-histochemical analyses were performed in joint tissue and distant organs (heart, spleen, kidney, liver and lung)	Ex vivo analysis was performed at 1 day, 1 week, 1, 2 and 6 months. Injected MSCs remained in the synovial cavity, engrafted within the cartilage lesion, and were detectable up to 1 month post-injection. No systemic distribution was observed, apart from 1 case of MSCs in the lung.	
Li et al. [67] (2016)	Mice	Osteoarthritis	Intraarticular (xenogenic MSCs—human MSCs)	MSCs were labeled with DiD fluorescent dye. In vivo bioluminescence imaging, and ex vivo quantitative PCR were performed to assess biodistribution.	Ex vivo imaging was performed up to day 70. PCR was performed at day 14 and 70 in heart, liver, spleen, lung, kidney, brain, muscle adjacent to the joint, and the whole injected knee join. MSCs were detected in the injected joint up to day 70 in diseased mice. In healthy mice, MSCs were detected up to day 21. No systemic distribution of MSCs was found.	MSCs seem to stand long times in the injected joint with no systemic distribution.
Marquina et al. [104] (2017)	Rats	Intraarticular chondrocyte trasplantation	Intraarticular, intravenous, intraperitoneal (allogenic MSCs)	MSCs were labeled with luciferase. Bioluminescence imaging was performed to assess biodistribution.	Imaging was performed at 2 h, 24 h, 2, 4 and 5 days. After intraarticular injection, no distribution of MSCs was detected. After intravenous injection, most MSCs were trapped in the lungs and disappeared within 24 h. After intraperitoneal injection, MSCs were localized in the injection site without distribution up to 5 days.	
Li et al. [68] (2017)	Rats	Osteoarthritis	Intraarticular (xenogenic MSCs—human MSCs)	MSCs were labeled with DiD fluorescent dye. In vivo bioluminescence imaging and ex vivo histologic examinations were performed.	In vivo imaging was performed up to 70 weeks. MSCs were detected in the injected join up to 9 weeks. No systemic distribution was observed.	MSCs seem to stand long times in the injected joint with no systemic distribution.
Meseguer-Olmo et al. [21] (2017)	Rabbits	Healthy animals	Intraarticular and intravenous (xenogenic MSCs—human MSCs)	MSCs were labeled with^99m^Tc-HMPAO. Scintigraphic images and qPCR in tissues (liver, kidney, heart, lung, bladder, knee, gallbladder) were used for assessing biodistribution.	Images were taken every 30 s during 25 min. qPCR was performed at 24 h. Intravenous MSCs distributed mainly to the lungs. Intraarticular MSCs did not distributed.	
Toupet et al. [66] (2015)	Mice	Osteoarthritis and arthritis (unknown number)	Intravenous and intraarticular (xenogenic MSCs—human MSCs)	Human MSCs were infused, Quantitative assays for human DNA and mRNA were used to evaluate the distribution in 13 different organs.	Necropsies were performed at different times (1, 10, 30, 42) and PCR was performed. After intravenous infusion, MSCs were only detected in lungs in day 1. No MSCs were detected in day 10. After intra-articular injection, MSCs were detected for at least 10 days in osteo-arthritic knee joints. No MSCs were detected in other organs after in these mice.	After intra-articular injection, MSCs do not seem to distribute to other organs or tissues.
Shim et al. [73] (2015)	Mice	Osteoarthritis and healthy models	Intraarticular and intravenous (xenogenic MSCs—human MSCs)	Human MSCs were injected and qPCR tests were used to assess biodistribution in the different organs.	At 15 min and 8 h after injection, samples were collected from eight organs (spleen, kidney, liver, lymph nodes, muscle, lung, heart, brain). Blood concentrations were also monitored. After intravenous injection MSCs were detected immediately in blood, with a progressive decrease. After intraarticular injection, MSCs were detected in blood with a peak at 8 h. No systemic distribution was observed after intraarterial delivery. After intravenous injection, most MSCs were trapped in the lungs.	After intraarterial injection, MSCs are detectable in blood with a peak at 8 h. However, no systemic distribution is observed.
Delling et al. [69] (2015)	Sheep	Osteoarthritis	Intraarticular (autogenic MSCs)	MSCs were labelled with SPION particles. MRI and histological analyses were performed.	MR images were acquired at injection and at 1, 4, 8, and 12 weeks. Ex vivo histological examination was performed at 12 weeks. MSCs were found in the joint up to 12 weeks, without systemic distribution.	
Ikuta et al. [76] (2015)	Rats	Healthy and cartilage defect models	Intraarticular (a magnet was used for selective accumulation of MSCs) and intravenous (xenogenic MSCs—human MSCs)	MSCs were labeled with DiR fluorescent dye and iron nanoparticles. MRI and fluorescent imaging were used to assess biodistribution. Histological exams were also performed.	Bioluminescence imaging was performed immediately and 1, 3, 7, 14, 21, and 28 days after cell transplantation. At day 28, organs were collected for ex vivo analyses. After intraarticular injection, MSCs remained in the joint. The use of the magnet led to magnetic MSCs accumulation in the target lesion.	The use of a magnet during magnetic-labeled MSCs transplantation can lead to selective accumulation of cells into the cartilage defects.

**Table 5:** Biodistribution after intralesional administration (except for intra-central nervous system) of MSCs in animal models.

Article	Model	Disease (Number of Animals)	Route of Administration (Source of Cells)	Cell-Marking Technique	Detection Time and Outcome	Comments
Dave et al. [54] (2017)	Mice	Chronic bowel inflammation	Intra-cardiac (xenogenic MSCs—human MSCs)	MSCs were labeled with luciferase and red fluorescent protein. In vivo and ex vivo bioluminescence and histologic examinations were performed to assess biodistribution.	Images were taken up to 24 h after injection. Histology was performed at 24 h post injection. MSCs in healthy mice distributed mainly to lungs, spleen and liver. In contrast, MSCs in diseased mice were located mainly in the intestine, with low pulmonary captation.	After intracardiac injection, MSCs are able to distribute mainly to the inflamed intestine.
Jiang et al. [109] (2018)	Rats	Myocardial infarction model (repeated ischemia model)	Intra-myocardial (allogenic MSCs)	MSCs were harvested from male rats and injected into female rats. qPCR was performed in different tissues to assess biodistribution (heart, lungs, spleen and liver)	Examinations were performed 3 weeks after injection. MSCs had a greater homing in heart and a lower distribution to peripheral organs when repeated ischemia was applied.	
Bansal et al. [108] (2015)	Mice	Healthy model	Intra-myocardial and intravenous (allogenic MSCs and xenogenic MSCs—Human MSCs)	MSCs were labeled with ^89^Zr-desferrioxamine. PET scans and radioactivity analyses were performed to assess biodistribution	PET was performed at days 2, 4, and 7. Ex vivo radioactivity analyses were performed at day 7. After intra-myocardial injection, MSCs were retained in the myocardium, as well as redistributed to the lung, liver, and bone. Intravenously administered MSCs also distributed primarily to the lung, liver, and bone.	
Blazquez et al. [107] (2015)	Pigs	Myocardial infarction model	Intrapericardial (allogenic MSCs)	MSCs were labelled with SPION particles. Biodistribution was assessed with MRI, histology and PCR.	MRI was performed at days 3, 5 and 7. MSCs were detected to home mainly in the left ventricle. They were also detected in the right ventricle, and both atriums.	After intrapericardial injection, MSCs distribute mainly to left ventricle.
Lebouvier et al. [78] (2015)	Pigs and mice	Osteonecrosis of the femoral head	Intraosseous (xenogenic MSCs—Human MSCs)	Human MSCs were injected and qPCR, cytometry and histologic analysis was performed to assess biodistribution in different tissues (Femoral head, adyacent tissues, liver, kidneys, spleen, and lungs).	Tissues were collected at either 30 min or 24 h after injection. No MSCs were detected in other organs apart from the injection site.	
Khan et al. [71] (2018)	Mice	Tendon injury	Intralesional (autogenic MSCs)	MSCs were labelled with fluorescent-conjugated magnetic iron-oxide nanoparticles (MIONs) and were tracked with MRI, histology and flow cytometry.	Tendons were recovered post mortem at 1 day, and 1–2, 4, 12 and 24 weeks after MSC injection. MSCs distributed throughout the tendon synovial sheath but restricted to the synovial tissues, with no MSCs detected in the tendon or surgical lesion. After day 14, no MSCs were detected.	
Burk et al. [72] (2016)	Horse	Tendon injury	Intralesional (autogenic MSCs)	MSCs were 10106 Molday ION Rhodamine B-labeled. Biodistribution was assesd with MRI, flow cytometry and histology	Tracking techniques were performed up to 24 weeks after injection. Labeled cells could be traced at their injection site by MRI as well as histology for the whole follow-up period of 24 weeks. Furthermore, small numbers of labeled cells were identified in peripheral blood within the first 24 h after cell injection and could also be found until week 24 within the contralateral control tendon lesions that had been injected with serum	
Ryska et al. [106] (2017)	Rats	Fistula model in Crohn’s disease.	Intralesional (perifistula) (allogenic)	MSCs were labeled with luciferase. Bioluminescence imaging was performed to assess biodistribution.	Imaging was performed at days 0, 2, 7, 14 and 30. MSCs distributed only in the injection site, with a high reduction of luminescence by day 2. MSCs were detectable up to day 30.	No systemic distribution was shown after intralesional injection.
Zhu et al. [82] (2015)	Rats	Ovarian injury	Intraovaric and intravenous (xenogenic MSCs—Human MSCs)	MSCs were fluorescent labeled with PKH26. Ex vivo bioluminescence techniques were used to assess biodistribution (brain, liver, kidney, urocyst, ovary and uterus were collected).	Bioluminescence was performed 1, 15, 30 and 45 days after injection. After intraovaric injection, MSCs were detected only in ovaries and uterus. After intravenous injection MSCs were detected in liver, kidney, ovary and uterus.	
Sadeghi et al. [79] (2016)	Rats	Birth-trauma injury (urinary disfunction) (285 animals)	Intraurethral and intravenous (xenogenic MSCs—Human MSCs)	Alu genomic repeat staining, PKH26 labeling, and luciferase-expression labeling. Histologic, genetic and bioluminescence tests were performed to evaluate MSCs distribution.	Different assessments were performed at 0, 1, 4 and 10 days after injection. No positive Alu-stained nuclei were observed in urethras at 4, 10, and 14 days. PKH26-labelled cells were found in all urethras at 2 and 24 h. Bioluminescence study showed increased luciferase expression from day 0 to 1 following injection, with a progressive disappearance until day 7.	No MSCs were detected in periurethral tissue after intravenous injection. MSCs were detected for less than 7 days in periurethral tissues after local injection.
Li et al. [136] (2017)	Rabbits	Chronic salpingitis model	Intrauterus and intravenous (xenogenic MSCs—Human MSCs)	MSCs were labeled with green fluorescent protein and cyto-keratin 7. Ex vivo bioluminescence imaging was performed in different organs (oviduct, uterus, liver, and bladder).	The assessment was performed 1 week after perfusion. No clear results are derived from this study. MSCs were detected in the uterus, bladder and oviduct.	
Ryu et al. [80] (2018)	Sprague-Dawley rats	Interstitial cystitis/bladder pain sindrome (unknown number)	Injection into the outer layer of the bladder. (xenogenic MSCs—Human MSCs)	Genetic transduction with green fluorescent protein was wed for labelling. Longitudinal microcystoscopy (combining confocal microscopy and cystoscopy) was used to assess the distribution of MSCs.	Images were obtained between 3 and 42 days after transplantation. The number of cells detected decreased rapidly until day 7 and later decreased gradually until day 42. After day 30, MSCs migrated from the serosa and muscularis layers to the urothelium. At day 30, most of the cells were distributed in vascular structures.	MSCs are capable of migrating through the layers of the bladder and might be able to differentiate into perivascular cells after day 30 post injection.
Dou et al. [81] (2019)	Rats	Erectile dysfuncion (unknown number)	Intra-cavernosal. (xenogenic MSCs—Human MSCs)	MSCs were labelled with mKATE and Renilla reniformis luciferase. Bioluminescence was used to assess the biodistribution. Histologic samples were obtained from penis, kidney, liver, lung, heart, skin, prostate, testis and spleen.	Bioluminescence was performed immediately after injection and up to 60 min. Histologic samples were obtained at days 1, 3 and 7 after injection. In vivo, MSCs immediately distributed in the para-penile region. An early migration to the abdominal area was noted, where the cells remained up to day 1. Histologic examinations showed MSCs in the penile, kidney, prostate and hepatic tissues.	Bioluminescence might be less sensitive to detect MSCs in distant tissues.
Kallmeyer et al. [114] (2020)	Rats	Cutaneous wound	Intradermal and intravenous (allogenic MSCs)	MSCs were labeled with luciferase and green fluorescent protein. Bioluminescence imaging and immunohistological analysis were performed to assess biodistribution.	Imaging was performed at 3 h, 24 h, 48 h, 72 h and 7 and 15 days. Intravenous MSCs were detected in the lungs 3 h after injection with a signal disappearance from 72 h. No MSCs were detected in the wound. Locally administered MSCs remained strongly detectable for 7 days at the injection site without systemic distribution.	
Tappenbeck et al. [112] (2019)	Mice	Healty animals (unknown number)	Intradermal and intravenous (xenogenic MSCs—Human MSCs)	Human MSCs were injected and genetic tests (quantitative PCR) were done in tissue samples: blood, skin/subcutis and skeletal muscle at the injection site, lymph node, liver, spleen, lungs, brain, femur bone, and bone marrow, kidneys, thymus, thyroid/para-thyroid gland and ovaries or testes) to evaluate biodistribution.	After intradermal injection, mice were sacrificed at 1 week, 3 months and 4 months. After intravenous injection, mice were sacrificed. After intradermal injection, MSCs were detected in the skin up to 3 months and also in draining limph nodes after 1 week. No MSCs were detected in any other tissues. After intravenous injection, MSCs were detected mainly in the skin and muscle near to the injection site and also in the lungs on day 8. After 1 month, most MSCs were in the lungs. MSCs were also detected in low quantities in kidney and thymus after 1 month.	After intradermal injection, MSCs seem to remain in the skin and migrate to lymph node, without significant systemic distribution.
Zhou et al. [137] (2017)	Mice	Immune deficient mice	Intradermal (a slice of cells). (xenogenic MSCs—canine MSCs)	MSCs were labeled with ultrasmall super-paramagnetic Fe_3_O_4_ nanoparticles (USPIO). MRI was used to assess biodistribution.	MRI was performed at 1 week, 4 weeks and 12 weeks after transplanting the cell sheets. MSCs were detected up to 12 weeks with gradual decrease of the captation.	
Pratheesh et al. [113] (2017)	Rabbits	Cutaneous wound	Intradermal (xenogenic MSCs—goat MSCs)	MSCs were labeled with PKH26. Fluorescent microscopy was performed to assess biodistribution within the wound.	Skin samples were collected from respective wounds on 3, 7, 10 and 14 days. MSCs demonstrated a diffuse pattern of distribution initially and were later concentrated towards the wound edges and finally appeared to be engrafted with the newly developed skin tissue.	
Léotot et al. [77] (2015)	Mice	Immunodeficient mice	MSCs were pre-loaded into the bone graft (xenogenic MSCs—Human MSCs)	Human MSCs were used and qPCR tests were used to assess biodistribution.	Constructs and organs (liver, spleen, lungs, heart, and kidneys) were harvested 24 h or each week between 1 and 7 weeks after implantation procedures. No biodistribution of MSCs was detected. MSCs were detectable in the graft up to 6 weeks.	
Lopez-Santalla et al. [103] (2017)	Mice	Colitis	Intranodal injection (inguinal nodes) (xenogenic MSCs—Human MSCs)	MSCs were labeled with luciferase. Biodistribution was assessed with bioluminescence imaging.	Bioluminescence imaging was performed 48 h after injection. MSCs mainly remained in the injected lymph nodes or fat surrounding them 48 h after injection. No significant systemic distribution was found, although the amount of MSCs in the intestine was relatively high.	After intranodal injection, most MSCs remained in the injection site 48 h later.
Packthongsuk et al. [105] (2018)	Pigs	Healthy animals	Intraperitoneal (autogenic MSCs)	MSCs (in this case, isolated from Wharton’s Jelly) were labeled with SRY sequences and PKH26-labeled Ex vivo evaluation was performed with qPCR and confocal microscopy. Tissues were collected from the heart, lung, pancreas, liver, kidney, omentum, stomach, intestine, uterine horn, ovary, muscle, and bone marrow.	Biodistribution was assessed at 6 h, 24 h, and 7, 14 and 21 days after administration. All tissues were positive for MSCs for 1-day-, 1-week-, 2-week-, and 3-week-old recipients.	MSCs-injected IP consistently reached tissues throughout the body. This result indicates that intaperitoneal injection should be considered in MSCs transplantations.
Hsu et al. [102] (2017)	Mice	Severe combined immunodeficiency	Intrahepatic and intrasplenic (xenogenic MSCs—Human MSCs)	MSCs were labeled with luciferase, red fluorescent protein and herpes simplex virus-1 thymidine kinase. PET, CT, bioluminescence imaging and histological analyses were performed to assess biodistribution.	Images and ex vivo analysis were collected for weeks 1 to 4. The intrahepatic group showed a confined signal at the injection site, while the intrasplenic group displayed a dispersed distribution at the upper abdominal liver area, and a more intense signal.	
Liu et al. [100] (2017)	Mice	Healthy and NK-activated mice (unknown number)	Intrahepatic (xenogenic MSCs—Human MSCs)	MSCs were labeled with the Luc2-mKate2 dual-fusion reporter gene. Bioluminescence was performed to assess biodistribution.	Images were collected at multiple time points. Bioluminescence imaging showed a gradual decline in the signal in the liver in both groups. NK-activated group showed a significantly more rapid decrease in the signal.	NK cells seem to have a role in the elimination of MSCs transplanted into the liver.
Xie et al. [99] (2019)	Rats	Acute liver injury (unknown number)	Intrahepatic (xenogenic MSCs—Human MSCs)	MSCs were transduced sith hHNF4α and luciferase2-mKate2 genes. Bioluminescence imaging was used to track their biodistribution.	Imaging was performed immediately after transplantation and until disappearance of cells. MSCs were only distributed in the liver. They were cleared within a short time after transplantation.	
Yaochite et al. [101] (2015)	Mice	Stretozotocin-induced diabetes mellitus (unknown number)	Intrapancreatic and intrasplenic (allogenic MSCs)	MSCs were labelled with d-luciferin. Bioluminescence imaging techniques were used to assess the biodistribution.	In vivo analysis was performed 0, 1, 3, 5, 8 and 11 days after injection. Ex vivo analysis were performed 2 days after injection. Intrasplenic MSCs were retained in the spleen and distributed to the liver, with a progressive decrease up to 8 days. Intrapancreatic MSCs did not distribute to other organs, and had a progressive decrease up to 8 days.	Instrasplenic MSCs are capable of distribute to the liver. Intrapancreatic MSCs do not seem to be able to distribute to other organs.
Lopez-Santalla et al. [103] (2018)	Mice	Colitis	Intraperitoneal (xenogenic MSCs—Human MSCs)	MSCs were labeled with luciferase. Bioluminescence was used to assess the biodistribution.	Biodistribution of MSCs was measured in the main organs (liver, spleen, intestine, lungs, heart and blood) and lymph nodes (LNs, inguinal, popliteal, parathymic, parathyroid, mesenteric, caudal and axillary) 48 h after injection. Most MSCs distributed to abdominal organs (liver, spleen and intestine), with few remaining in lymph nodes, lungs, blood and heart. Biodistribution did not change significantly between healthy and diseased mice.	Intraperitoneal injection seems to lead to abdominal spreading of MSCs.
Chen et al. [110] (2020)	Rats	Broncopulmonary dysplasia	Intratracheal (xenogenic MSCs—Human MSCs)	MSCs were labeled with Green Fluorescent Protein and luciferase. Bioluminescence was used to assess the biodistribution in the lungs.	Images were taken every 5 s up to 1 min. MSCs distributed in the lungs without systemic distribution.	Intratracheal injection lacks systemic distribution of MSCs.
Cardenes et al. [111] (2019)	Sheep	Acute respiratory syndrome	Intrabronchial and intravenous (xenogenic MSCs—Human MSCs)	MSCs were labeled with ^18^FDG. PET-TC was performed to assess biodistribution.	Images were taken 1 and 5 h after cell administration. After intrabronchial administration, MSCs remained in the injection site at 1 and 5 h without systemic distribution. After intravenous injection, MSCs distributed widely to organs, but with a preference for the lungs.	Both administration routes are convenient for treating acute respiratory syndrome.

**Table 6:** Biodistribution after intra-central nervous system administration of MSCs in animal models.

Article	Model	Disease (Number of Animals)	Route of Administration (Source of Cells)	Cell-Marking Technique	Detection Time and Outcome	Comments
Li et al. [98] (2015)	Different animals and models (the article is a review)		Intranasal (different sources)	Different techniques.	Some results are: MSCs reached an intracerebral glioma site within 6 h after i.n. delivery, with a further significant increase in cell numbers within 24 h; Intranasal application of MSCs resulted in the appearance of cells in the olfactory bulb, brain and spinal cord, and about one-fourth of MSCs survived for at least 4.5 months in the brain.	
Zhang et al. [138] (2017)	Rats	Spinal cord injury	Intra-spinal cord (allogenic MSCs)	MSCs were labeled with Gd-DTPA-FA and neurofilament-200. MRI and histological examinations were performed to assess biodistribution.	Examinations were performed at day 1, 7, 14 and 28 post delivery. In the first 7 days, transplanted cells were observed near the injection point. The number of cells reached a maximum at day 14 and then gradually distributed along the segmental injury. No systemic distribution was observed.	
Barberini et al. [83] (2018)	Horses	Healthy and myelopathy-model animals. (9 animals)	Intrathecal, both atlanto-occipital (AO) and lumbo-sacral (LS) injection. (allogenic MSCs)	^99m^Tc-HMPAO was used to label MSCs. Later evaluation was performed with a gamma camera and histologic samples.	Imaging was performed each hour until 5 h post-infusion. MSCs administered by AO injection were found to distribute caudally through-out the vertebral canal. MSCs administered by LS injection did not distribute cranially. Histologic tests did not show the presence of MSCs in diseased areas.	LS injection of MSCs does not seem to be proper to treat central nervous system distant lesions.
Quesada et al. [85] (2019)	Mice	Non-obese diabetic severe combined immunodeficiency mice	Intrathecal (xenogenic MSCs—Human MSCs)	Human MSCs were used. Histologic evaluation and qPCR were performed in different tissues (Heart, brain, cerebellum, spinal cord, liver, spleen, lungs, kidneys and gonads).	Evaluation was performed 24 h and 4 months after injection. 24 h post-injection, MSCs were detected in the spinal cord and in 1 heart. 4 months after injection, MSCs were detected in 3 hearts and in 1 brain.	
Kim et al. [84] (2020)	Rats	Healthy rats	Intrathecal (injected via L2-L3 space) (xenogenic MSCs—Human MSCs)	Fluorescent dye DiD was used to label MSCs. Ex vivo bioluminescence and qPCR of brain, spine and heart, lung, liver, spleen, and kidney was used to assess biodistribution.	Imaging was performed at 0, 6, and 12 h post injection. MSCs were detected in the spinal cord at all times. MSCs were found in the brain only at 12 h. No other organs showed MSCs. Increasing the Cell Injection dose of MSCs improved the migration of MSCs to the brain.	MSCs are able to migrate from spinal cord to the brain. This migration can be improved by the increase of the dose.
Violatto et al. [97] (2015)	Mice	Amyotrophic lateral sclerosis model	Intracerebral (lateral ventricles) and intravenous (xenogenic MSCs—Human MSCs)	MSCs were double labeled with fluorescent nanoparticles and Hoechst-33258. Bioluminescence and histologic examinations were used to assess biodistribution.	In vivo and ex vivo analyses were performed at 1, 7, 21 days. By intravenous administration cells were sequestered by the lungs and rapidly cleared by the liver. MSCs transplanted in lateral ventricles remained on the choroid plexus for the whole duration of the study even if decreasing in number. Few cells were found in the spinal cord, and no migration to brain parenchyma was observed	
Geng et al. [91] (2015)	Rats	Cerebral ischemia	Intracerebral (allogenic MSCs)	A gadolinium-based cell labeling technique was used. MRI images were used to assess biodistribution.	MRI was used to image the cells 1,3, 5 and 7 days after the Gd-MSC injection. MSCs did not distribute systemically.	
Mastro-Martinez et al. [90] (2015)	Rats	Traumatic brain injury	Intracerebral (allogenic MSCs)	Green fluorescent protein was used to label cells. Histological examinations and immunochemistry were used to assess biodistribution.	Histologic examination was performed at 24 h and 21 days after transplantation. MSC were found in the perilesional area at 24 h, and their number decreased with time after transplantation. MSC treatment increased the cell density in the hippocampus and enhanced neurogenesis in this area.	
Park et al. [96] (2016)	Beagle dogs	Healthy animals	Intracerebral (intra-ventricular) (xenogenic MSCs—Human MSCs)	Human MSCs were used. Immunohistochemical and qPCR were performed to assess biodistribution.	Brains were collected 7 days after infusion. MSCs migrated from ventricles towards the cortex, being found in the brain parenchyma, especially along the lateral ventricular walls. MSCs were also detected in the hippocampus and the spinal cord. No systemic distribution of MSCs was detected.	
Xie et al. [87] (2016)	Rats	Intracerebral hemorrhage	Intracerebral and intravenous (xenogenic MSCs—Human MSCs)	A fluorescent dye was used to label MSCs (CM-DiI). Histologic evaluation was used to assess distribution of MSCs.	Histologic examination was performed at 28 days. After intracerebral injection, MSCs stayed in the injection place, distributed around the hemorrhage. A small amount of cells migrated to the contralateral hemisphere. After intravenous injection, MSCs were also found in the cerebral area.	Both intracerebral and intravenous routes are appropriate for treating intracerebral hemorrhage.
Duan et al. [88] (2017)	Rats	Cerebral ischemia (54 animals)	Intracerebral injection (right striatum)	Green fluorescent protein MSCs (GFP-MSCs) and SPION labeled. MRI and histology were used to assess biodistribution.	Imaging and/or histology were performed weekly from week 1 to 8 weeks after cells transplantation. MSCs were found to remain in the area in a high quantity in week 1. Later, MSCs number decreased drastically, being detectable up to week 8. A small amount of cells migrated to corpus callosum.	
Dong et al. [95] (2017)	Rats	Brain traumatic injury (30 animals)	Intracerebral injection (intraventricular) (allogenic MSCs)	Green fluorescent protein MSCs (GFP-MSCs). Imaging techniques and histology were used to assess biodistribution in blood vessels.	Techniques were performed at 10, 14, and 17 days. MSCs were found to home in large arteries (thoracic aorta, abdominal aorta, common iliac artery) 10, 14, and 17 days after transplantation.	MSCs seem to distribute after brain injury when injected intraventricularly.
Lee et al. [89] (2017)	Mice	Familial Alzheimer’s disease	Intracerebral injection (Injection into the hippocampi) (xenogenic MSCs—Human MSCs)	Ferumoxytol was used to label MSCs. MRI and histology were used to assess biodistribution.	Techniques were performed at 1, 7 and 14 days. MSCs were found to remain in the injection site up to 14 days after injection.	
Wang et al. [86] (2018)	Sprague Dawley rats	Glioma (unknown number)	Intracerebral (MSCs were injected contralaterally to glioma) (allogenic MSCs)	CM-Dil staining was used to label MSCs, which also contained Paclitaxel. Confocal laser-scanning microscopy was used to assess the distribution of MSCs. Later histological examinations assessed the distribution of MSCs within the brain.	Necropsies were performed 2 days after MSCs injection. MSCs were distributed in clusters in the injection area, and were also found within the glioma.	MSCs seem to spread within a short period of time from one hemisphere to another, after intracerebral injection.
Mezzanote et al. [94] (2017)	Mice	Healthy mice (unknown number)	Intracerebral injection (brain cortex)	MSCs were transfected with a novel bioluminescent/near infrared fluorescent (NIRF) fusion gene. Fluorescence images and bioluminescence were used to assess the distribution of the cells.	Images were taken up to week 7 after transplantation. Movement of the MSCs was not assessed. MSCs were detected for 7 weeks without a significant drop in bioluminescent signals, suggesting the sustained viability of hMSCs transplanted into the cortex.	No specific biodistribution assessment.
Da Silva et al. [92] (2019)	Rats	Ischemic stroke model	Intracerebral injection (xenogenic MSCs—Human MSCs).	MSCs were labeled with luciferase and multimodal nanoparticles with iron. In vivo bioluminescence, near-infrared imaging and ex vivo MRI were used to assess biodistribution.	Biodistribution was assessed at 4 h and 6 days after cell injection. MSCs did not distribute. The amount of MSCs decreased drastically from 4 h to 6 days.	
Ohki et al. [93] (2020)	Rats	Healthy model	Intracerebral injection (xenogenic MSCs—Human MSCs).	MSCs were labeled with SPIO or USPIO. MRI and histological examinations were performed to assess biodistribution.	MRI images were obtained immediately and at 7- and 14-days post injection. No MSCs demonstrated migration.	
Sukhinich et al. [53] (2020)	Rats	Healthy model	Intracerebral and selective intra-arterial (internal carotid artery) (xenogenic MSCs—Human MSCs).	MSCs were labeled with SPION and PKH26. MRI imaging and histology were performed to assess biodistribution.	The distribution and migration of MSC were analyzed by MRI from day 1 to day 15, and histological methods on days 1, 2, 3, 7, and 15. After intracerebral injection, MSCs moved to corpus callosum and blood vessels. After intraarterial injection, most MSCs were detected in the ipsilateral hemisphere and most of them within the blood vessels.	
Teo et al. [139] (2015)	Mice	Dermal inflammation (unknown number)	Retro-orbital injection. (xenogenic MSCs—Human MSCs).	MSCs were labelled with specific techniques for intravital confocal microscopy (DiI, DiO, DiD or DiR solution). Later confocal microscopy was used to assess the histologic distribution of MSCs	Imaging was performed 2 h, 4 h and 6 h after the MSC had been infused. When MSCs were detected, images were taken every 5 min. By 2 h post-infusion, arrested and transmigrating MSC were equally distributed within both small capillaries and larger venules. These MSCs were in contact with neutrophil-platelet clusters. Platelet depletion led to significantly reduced the preferential homing of MSC to the inflamed	Authors concluded that MSCs transmigrate to tissues due to the existence of an active adhesion mechanism. Platelets seem to play a crucial role in MSCs trafficking.

**Table 7:** Articles regarding biodistribution of MSCs in humans.

Article	Disease (Number of Patients)	Route of Administration (Source of Mscs)	Cell-Marking Technique	Detection Time and Outcome	Comments
Krueger et al. [126] (2018)	Breast cancer (28 patients) [116]	Intravenous (autogenic MSCs)	Flow citometry	MSCs were detected for several hours post-infusion in peripheral blood. MSCs are rapidly (less than 1 h) cleared from peripheral blood after intravenous infusion	The presence of MSCs in peripheral blood was not detected after 1 h post-infusion.
	Cirrhosis (4 patients) [115]	Intravenous (autogenic MSCs)	^111^In-oxine labeled mesenchymal stem cells, evaluated with Dual head gamma camera and SPECT imaging techniques.	MSCs were detected at 2 h, 4 h, 6 h, 24 h, 48 h, 7th and 10th days after infusion. Pre-48 h images showed a large majority of cells distributed in the lungs. Later images showed a drastic decrease in lung captation, with a higher amount of MSCs distributed in the spleen and few in liver.	There was a clear initial biodistribution in lungs, which decreased after 48 h. Spleen captation was higher than liver captation, maybe due to splenomegaly.
Henriksson et al. [118] (2019)	Intervertebral disc degeneration (4 patients)	Intralesional (autogenic MSCs)	MSCs were labeled with iron sucrose (Venofer^®^). Histologic examinations were performed to detect the cells.	Intravertebral discs were explanted at 8 months (3 patients) and 28 months (1 patient) post injection. MSCs were detected at 8 months, but not at 28 months. Detected MSCs had differentiated into chondrocyte-like cells.	MSCs seem to home in intravertebral discs after intralesional injection for long periods of time.
Sokal et al. [117] (2017)	Haemophilia A (1 patient)	Intravenous (Adult-derived human liver stem cells) (allogenic MSCs)	MSCs were labeled with ^111^In-Oxine and biodistribution was assessed with sequential planar imaging (SPECT, TC).	Total body imaging was performed at 1, 4, 24, 48, 72, and 144 h postinfusion. MSCs were initially (1 h) trapped in the lungs and liver. At 24 h, MSCs were detected in the right ankle (where hemarthrosis was recurrent). Up to day 6, lungs signal decreased and liver signal increased. MSCs were also detected in small amounts in spleen and bone marrow.	MSCs infusion seemed to result in an improved bleeding phenotype and was well tolerated. Moreover, the distribution of MSCs to the place of bleeding suggests possible in situ production of factor VIII.
Sood et al. [122] (2015)	Type 2 diabetes mellitus (21 patients)	Intravenous and selective intraarterial (superior pancreaticoduodenal artery and proximal splenic artery) (autogenic bone marrow MSCs).	MSCs were labeled with 18-FDG. PET-TC images were used to assess biodistribution	Images were taken at 30 and 90 min post infusion. In the intravenous group, MSCs distributed to lungs at 30 min with significant clearance in the delayed 90-min image, with no distribution to pancreas. Selective intraarterial delivery led to MSCs homing in pancreas head (pancreaticoduodenal artery) or body (splenic artery).	Selective intraarterial delivery leads to selective homing of MSCs into the pancreas.
Lezaic et al. [119] (2016)	Idiopathic dilated cardiomyopathy (35 patients)	Intracoronary infusion (autogenic MSCs)	MSCs were labeled with ^99m^Tc-HMPAO. Gamma-cammera images were taken to assess biodistribution.	Imaging was performed 1 h and 18 h after transplantation. At 1 h after intracoronary administration, the majority of MSCs accumulated in the liver, spleen and bone marrow. Accumulation of MSCs in the myocardium ranged from 0 to 1.45% of injected activity in the field of view. The distribution of labeled stem cells in the myocardium corresponded to the area supplied by the vessel used for administration. At imaging 18 h post injection, the distribution of labeled stem cells appeared unchanged, but with decreased activity.	The retention of MSCs in the myocardium is low after intracoronary injection.

## Figures and Tables

**Table 1:** Overview of the characteristics of each route of administration.

Route of Administration	Systemic Distribution	Organs to Which the Cells are Distributed	Advantages	Disadvantages
Intravenous	Yes	First, cells move to the lungs (first capillary filter). Later, cells distribute, mainly to the liver, spleen and kidneys. Variable amounts of cells are found in other organs.	Convenient route of administration. Widely used. Useful to reach the lungs.	Cells do not reach other organs apart from lungs in great quantities.
Intraarterial (not selective)	Yes	Cells bypass the pulmonary filter so there is a wide distribution in the rest of the organs (heart, brain, kidneys, liver, digestive system)	Convenient route of administration. Useful to bypass the lungs and achieve broader distribution.	Not so widely used. Intraarterial infusion is not common in clinical practice
Intraarterial (selective)	Yes (reduced)	Cells are distributed mainly in the territory irrigated by the cannulated artery. Distribution of cells to other organs is possible but in smaller amounts.	Targeted deposition of cells is achieved.	Inconvenient route of administration. Difficult to transfer to clinical practice
Intramuscular and intraarticular	No	Cells remain at the injection site	Convenient route of administration. Targeted deposition of cells is achieved	No systemic distribution is achieved.
Intradermal, intratracheal, intrapulmonary and intraurinary tissue	No	Cells remain at the injection site	Convenient route of administration, depending on each specific route. Targeted deposition of cells is achieved	No systemic distribution is achieved.
Intrahepatic, intrasplenic, intrapericardial, intramyocardial	Yes	Cells distribute following the direction of the bloodstream derived from the infused organ.	Targeted deposition is achieved. Knowledge about the bloodstream derived from the infused organ might lead to targeted distribution after injection.	Inconvenient in clinical practice. Difficult to transfer to clinical practice.
Injection into cavities containing body fluids (peritoneum, cerebral ventricles)	Yes (low amounts)	Cells distribute mainly to tissues in contact with the body fluid.	Convenient route of administration, depending on each specific route. Targeted deposition is achieved. Limited systemic biodistribution.	Limited systemic biodistribution.
Intrathecal administration	No	Cells distribute caudally when injected in the upper segments of spine. Cranial migration of cells after lumbar injection seems to be possible if a high dose of MSCs is administered (e.g., distribution to brain).	Convenient route of administration if deposition of the cells at the central nervous system level is desired. Limited systemic biodistribution.	Inconvenient in clinical practice (depending on the cases).
Intra-Central Nervous System	Yes/No (variable amounts)	Cells are able to distribute within the central nervous system. Factors leading to the movement of the cells are still not clear.	Targeted deposition is achieved.	Inconvenient route of administration. Difficult to transfer to clinical practice.

## Data Availability

Not applicable.
